# Porcine reproductive and respiratory syndrome virus antagonizes the host restriction factor SHFL to sustain viral programmed ribosomal frameshifting and replication

**DOI:** 10.1128/jvi.00119-26

**Published:** 2026-04-21

**Authors:** Kefan Bu, Jing Shi, Jingjing Ding, Tao Xu, Qingyu Li, Chenxi Li, Yanhua Li

**Affiliations:** 1 College of Veterinary Medicine, Yangzhou University, Yangzhou, Jiangsu, China; 2 Comparative Medicine Research Institute, Yangzhou University, Yangzhou, Jiangsu, China; 3 Jiangsu Co-innovation Center for Prevention and Control of Important Animal Infectious Diseases and Zoonoses, Yangzhou, Jiangsu, China; University of Kentucky College of Medicine, Lexington, Kentucky, USA

**Keywords:** PRRSV, programmed ribosomal frameshifting (PRF), SHFL, virus-host interaction

## Abstract

The global pandemic of porcine reproductive and respiratory syndrome virus (PRRSV) and its continuous evolution pose a sustained threat to the swine industry, underscoring the urgent need for a broad-spectrum antiviral strategy. Programmed ribosomal frameshifting (PRF) is an essential mechanism in PRRSV that directs the synthesis of viral nonstructural proteins (nsps) and represents an attractive antiviral target. Here, we show that SHFL, an interferon-stimulated gene (ISG), restricts PRRSV infection by specifically targeting viral biosynthesis steps. Mechanistically, SHFL inhibits the canonical programmed −1 ribosomal frameshifting (−1 PRF) of PRRSV, thereby suppressing RNA replication. SHFL also effectively blocks PRRSV −2 PRF, demonstrating its broad-spectrum activity against different frameshifting mechanisms. To sustain viral replication, PRRSV employs its nsp12 and nucleocapsid (N) proteins to counteract the expression of SHFL transcriptionally, thereby attenuating this host antiviral defense. These findings identify SHFL as a broad-spectrum PRF inhibitor against PRRSV, further elucidating its antiviral mechanisms of SHFL. The discovery that PRRSV proteins antagonize SHFL not only deepens our understanding of PRRSV pathogenesis but also highlights the dynamic interplay between virus and host.

**IMPORTANCE:**

Porcine reproductive and respiratory syndrome virus (PRRSV) is a globally prevalent and economically devastating swine pathogen. Due to the high genetic diversity of PRRSV strains in the field, the current vaccination strategy cannot provide enough protection against PRRS threats. Programmed ribosomal frameshifting (PRF) is an essential mechanism in PRRSV that directs the synthesis of viral nonstructural proteins (nsps) and represents an attractive antiviral target. In this study, we conducted a comprehensive investigation into the antiviral effect of SHFL on PRRSV infection. Both canonical −1 PRF and the novel −2 PRF of PRRSV are suppressed by SHFL, demonstrating SHFL’s broad-spectrum activity against different frameshifting mechanisms. We also found that PRRSV nsp12 and N counteract the expression of SHFL transcriptionally, thereby attenuating this host antiviral defense. Our study provides new insight into the antiviral mechanism of SHFL and the dynamic virus-host interaction, which may have implications for the development of PRRS control strategies.

## INTRODUCTION

Porcine reproductive and respiratory syndrome virus (PRRSV) is a globally prevalent and economically devastating swine pathogen. It primarily causes reproductive disorders in pregnant sows and respiratory diseases in pigs of all ages (1). PRRS imposes major economic losses on the swine industry worldwide, with estimated losses of around 1,424.37 yuan per sow in China, 126 euros in Europe, and 121 dollars in the United States (2). The high genetic diversity of PRRSV, driven by constant mutation and recombination, has hampered the development of effective vaccines (3). This underscores the need for more comprehensive strategies to address emerging threats.

PRRSV is an enveloped, single-stranded, positive-sense RNA virus belonging to the family *Arteriviridae* within the order *Nidovirales*. It has been classified into two species: PRRSV-1 (European genotype) and PRRSV-2 (North American genotype) (4). While PRRSV-2 has been the predominant species in China, PRRSV-1 has been increasingly isolated in recent years (5–8). The PRRSV genome is approximately 15 kb in length and comprises at least 11 open reading frames (ORFs), with a 5′-untranslated region (UTR) and a poly(A) tail following the 3′-UTR. ORF1a and ORF1b at the 5′ end encode two large replicase polyproteins, pp1a and pp1ab. The expression of pp1ab depends on a −1 ribosomal frameshifting signal at the ORF1a/ORF1b overlapping region. These polyproteins are subsequently processed into at least 14 nonstructural proteins (nsps). Notably, PRRSV nsp2TF and nsp2N are translated through a unique programmed −2/−1 ribosomal frameshifting (−2/−1 PRF) in the nsp2-coding region (9, 10). A previous study indicates that nsp2TF is significant for PRRSV replication (11). In addition, both nsp2TF and nsp2N have been implicated in suppressing host immune responses (12–14). Furthermore, most of the nsps assemble into a replication and transcription complex (RTC), which directs the replication of the viral genome and the synthesis of a set of subgenomic RNAs (sgRNAs). These sgRNAs encode the eight different PRRSV structural proteins required for production of mature virions.

−1 PRF was first described in Rous sarcoma alpharetrovirus (15, 16). −1 PRF is a noncanonical translational strategy employed by many RNA viruses, where it controls the stoichiometry of viral proteins essential for viral replication and expands viral genomic coding capacity via overlapping ORFs (17, 18). Critically, alterations to the −1 PRF frequency can impair essential viral functions like hindering assembly and replication, making it a promising target for antiviral intervention (19–21). Canonical −1 PRF signals are characterized by two core elements, a heptanucleotide “slippery” sequence and a downstream RNA secondary structure spaced 5–9 nucleotides (nt) apart. This structure acts as a roadblock, pausing the elongating ribosome over the slippery sequence and then slipping backward by one nucleotide to continue translation in a new reading frame (22). PRRSV −1 PRF signal comprises a U_UUA_AAC slippery sequence and a predicted pseudoknot beginning 5 nt downstream, although the structure has not been verified by experiments. Rather than an RNA secondary structure, the stimulatory element of PRRSV −2/−1 PRF signal is a C-rich motif (CCCANCUCC) located 10 nt downstream of the slippery sequence (G_GUU_UUU). Furthermore, −2/−1 PRF requires a transactivation complex formed by host protein PCBP and the viral protein nsp1β (10, 23).

While the stability, plasticity, and unfolding kinetics of these cis-acting elements influence ribosomal frameshifting efficiency (24, 25), numerous trans-acting factors can also regulate PRF efficiency. In T cell lines, tRNA modification and abundance lead to stimulation of frameshifting (26). Studies have shown that the cardiovirus 2A protein can stabilize pseudoknots to promote −1 PRF (27), while the host factor eIF2A enhances SARS-CoV-2 −1 PRF by binding CG-rich motifs (28). Conversely, interferon-stimulated genes (ISGs) can act as potent suppressors, like SHFL and the short isoform of zinc-finger antiviral protein (ZAP-S), which directly bind the SARS-CoV-2 frameshift element to inhibit PRF (29–31). SHFL (formerly C19orf66, also named RyDEN, IRAV, FLJ11286, or shiftless) was first identified as an ISG in 2011 (32). It is highly conserved in vertebrates and has been established that there are two isoforms of SHFL arising from alternative splicing, including the long form comprising 291 amino acids, and the short form, namely SFLS, lacking 164th–199th amino acids compared with the long form (30). The long‐form SHFL protein contains eight α‐helices, seven β‐strands, and possesses a zinc-ribbon domain, a nuclear localization signal, a nuclear export signal, and a coiled‐coil motif. It also has a unique glutamic acid-rich (E-rich) domain in its C-terminus (33). SHFL has been reported as a broad-spectrum virus-inhibiting ISG, capable of suppressing the replication of multiple DNA and RNA viruses (30, 33–41). SHFL’s complicated and versatile structures and domains thus appear to mirror its diversity of antiviral functions, mainly focusing on inhibiting −1 PRF, degrading RdRp of viruses, and decreasing viral RNA expression levels (42). For example, the domain spanning 164th–199th amino acids is important for SHFL-mediated restriction of the −1 PRF, and it is required to restrict the replication of HIV-1 (30), SARS-CoV-2 (21), and Japanese encephalitis virus (JEV) (36). While the broad antiviral activities of SHFL are increasingly recognized, its specific molecular function in PRRSV infection is poorly understood. Hence, we focused our investigation on the potential antiviral effect of SHFL against PRRSV infection and the underlying mechanisms. In addition, although SHFL is known as a broad-spectrum inhibitor of −1 PRF, its ability to suppress −1 PRF of PRRSV as well as the novel −2/−1 PRF remains to be elucidated.

In this study, overexpression, gene knockdown, and knockout experiments revealed that SHFL confers resistance to all PRRSV species in both target and permissive cells. We further established that SHFL interferes primarily with viral RNA translation and replication. Mechanistically, we provide evidence that this inhibition is achieved by targeting PRF, both canonical −1 PRF and the novel −2/−1 PRF events. We identified the highly conserved −1 PRF signal of PRRSV, comprising a slippery sequence and a downstream pseudoknot whose structural stability is critical for frameshifting efficiency. Interestingly, we observed that PRRSV counteracts this inhibition, as PRRSV nsp12 and nucleocapsid (N) appear to downregulate the expression of SHFL at the transcription level. Collectively, our findings indicate that SHFL acts as an antiviral factor against PRRSV by mainly targeting PRF, and PRRSV has evolved mechanisms to partially evade this inhibition.

## MATERIALS AND METHODS

### Cell lines and cell culture

HEK-293T, BHK-21, and LLC-PK1 cells were purchased from American Type Culture Collection (ATCC) and cultured in Dulbecco modified Eagle medium (Cytiva) containing 10% fetal bovine serum (FBS, Sigma-Aldrich), 100 μg/mL streptomycin, and 100 IU/mL penicillin (Hyclone). MARC-145 cells purchased from the ATCC were cultivated in modified Eagle’s medium (MEM, Cytiva) containing 10% FBS (Sigma-Aldrich), 100 μg/mL streptomycin, and 100 IU/mL penicillin (Hyclone). Porcine alveolar macrophages (PAMs) isolated from the lung lavage of PRRSV-negative piglets were cultured in RPMI-1640 medium (Cytiva) containing 10% FBS (Sigma-Aldrich) and 100 μg/mL streptomycin and 100 IU/mL penicillin (Thermo Fisher Scientific). All cell cultures were maintained at 37°C in a 5% CO_2_ incubator (Thermo Fisher Scientific).

### Viruses

The NADC30-like PRRSV-2 NL1207 strain (GenBank accession no. MZ399800.1) and the PRRSV-1 SHE strain (GenBank accession no. GQ461593.1) were passaged in PAM or MARC-145 cells. The reporter virus TA-EGFP (rTA-EGFP) was engineered to harbor an EGFP gene in the nsp2-coding region and was passaged in MARC-145 cells. The reporter virus TA-NLuc (rTA-NLuc) was engineered to harbor an NLuc luciferase gene in the nsp2-coding region and was passaged in MARC-145 cells. Virus supernatants were collected, clarified by centrifugation, and stored at −80°C. Virus titration was performed using MARC-145 cells. Virus infections were performed at the indicated multiplicity of infection (MOI) and incubated at 37°C for 2 h. The unbound virus was removed by washing with phosphate-buffered saline (PBS). Cells were then maintained in MEM containing 2% FBS (Sigma-Aldrich), 100 μg/mL streptomycin, and 100 IU/mL penicillin (Thermo Fisher Scientific).

### Antibodies and reagents

The following antibodies purchased from commercial resources were used in this study, including anti-FLAG mouse monoclonal antibody (MBL Biotech), DDDDK-Tag rabbit mAb (ABclonal Biotech), RyDEN polyclonal antibody (Proteintech Biotech), rabbit polyclonal antibody against the PLP2 domain of PRRSV nsp2 generated by GenScript , mouse monoclonal antibody against STAT1 (Santa Cruz Biotechnology), histone H3 mouse monoclonal antibody (HUABIO Biotech), calnexin rabbit mAb (ABclonal Biotech), anti-β tubulin rabbit pAb (Bioworld Technology), HRP-conjugated goat anti-mouse and HRP-conjugated goat anti-rabbit antibodies (BBI Life Science), TRITC-conjugated goat anti-rabbit IgG and Alexa Fluor 488-conjugated anti-mouse antibody (Jackson ImmunoResearch), and mouse monoclonal antibody against PRRSV N (Keepseeking Biotech, Guangzhou, China). A rabbit polyclonal antibody against nsp4 was kindly provided by Dr. Yihong Xiao from Shandong Agricultural University. Monoclonal antibodies against nsp9 and nsp12 were kindly provided by Dr. Lei Zhou from China Agricultural University and Dr. Liwei Li from Shanghai Veterinary Research Institute, CAAS. The following reagents were used: puromycin (YEASEN Biotech), recombinant human interferon-alpha 2b (IFN-α2b) (GenScript Biotech, Nanjing, China), Polybrene (Solarbio), DAPI (10 μg/mL, Solarbio), coelenterazine h (Shanghai Maokang Biotechnology), pronase (Sigma-Aldrich), and furimazine (Boluteng, Guangzhou, China).

### Plasmid

As described in our previous study (10), a dual luciferase reporter system was used to assess the frameshift efficiency. To create a similar reporter plasmid, a 78-nt sequence covering the predicted −1 PRF RNA signal of PRRSV TA-12 strain was inserted between the Renilla luciferase (RLuc) and Firefly luciferase (FLuc) genes, with the downstream FLuc in the −1 frame of RLuc. For the in-frame control (IFC) construct, two mutations were introduced at the slippery sequence to make FLuc and RLuc in the same reading frame. The −1 PRF sequence was truncated to generate a panel of constructs to map the minimal RNA signal required. The PN1 and PN3 constructs containing partially or completely disrupted predicted RNA pseudoknot were generated. The pseudoknot was reconstituted through compensatory mutations in constructs PN2 and PN4. The dual luciferase reporter plasmid for PRRSV −2 PRF was created by cloning the −2 PRF sequence (79 nucleotides) of the PRRSV-2 TA-12 strain into the pSGDluc-V3.0 as described previously (10, 43). In this reporter plasmid, the downstream FLuc ORF is in the −2 frame of RLuc. The −2 PRF IFC control plasmid was generated by introducing mutations at the slippery sequence (10). The *Sus scrofa shfl* gene (NM_001244321) was codon optimized and synthesized by GenScript Biotech. The plasmid expressing pig SHFL was created by cloning the FLAG-tagged *shfl* gene into the pcDNA3.1(+) vector. A panel of constructs containing *shfl* mutants was generated, including three truncation mutants (1–260, SFLS, and Δ102–150), SHFL-Zincmut with the zinc finger domain mutated (36), and the 3R/A mutant containing R131A/R133A/R136A mutations to abrogate RNA binding (44). A series of truncated SHFL promoter fragments, amplified from LLC-PK1 genomic DNA, was cloned into the pGL3-Basic vector to generate luciferase reporter constructs for assessing promoter activity. Primers used for the generation of these plasmids are listed in Table 1, and all constructs were verified by Sanger sequencing.

**TABLE 1:** Primers for plasmid construction

Name	qPCR primer (5’−3’)
shfl-sgrna-F	AAAGGACGACGTCTCGGGTATCCTCCAAGAAGGCGGGTTTTAGAGCTAGAAATAGCA
shfl-sgrna-R	GCTATTTCTAGCTCTAAAACCCGCAGGGGTAAAGCAAAAACGAGACGTCGTCCTTTC
SHFL-F1	CTGGCTAGCGTTTAAACTTA
SFLS-R1	TGGTTGCGAAAGTTGTGCCTACACTTGGGGCA
SFLS-F2	CACAACTTTCGCAACCACACACACAGCTGCTCC
SHFL-R3	GTGCTGGATATCTGCAGA
SHFL-Zincmut-R1	TCAGCGGAGCTGGCGGCAAACTGCCGGTCC
SHFL-Zincmut-F2	CCGCCAGCTCCGCTGATCACGTGTGGTGGAGG
SHFL-Zincmut-R2	CTAGCCTTTCTGGCCCGGGACACCTCCTTTCT
SHFL-Zincmut-F3	GGGCCAGAAAGGCTAGGAAGCGGTACGAGC
SHFL-Δ102-105-F	CAGGAGGATGCCGAGTTCCACTGCCCCAAGTG
SHFL-Δ102-150-R	GAACTCGGCATCCTCCTGGGCTCTCTGGAACATC
SHFL-1-260-R	TGTGCTGGATATCTGCAGAATTCTCACAGGCCGCCCTGA
SHFL-R3-F	CCGCGTGCGCAAAGTGTGCGAAGCGGTACGAGCCAGTG
SHFL-R3-R	CTTCGCACACTTTGCGCACGCGGACACCTCCTTTCTCTG
luc-1PRF-78-F	GCAGTATTTCTACACCTCGAGGTGTTTAAACTGCTAGCCG
luc-1PRF-69-R	CATCTTCAATGGTGGCAGATCTTGTCTCAGTAACAACTAAGC
luc-1PRF-72-R	CATCTTCAATGGTGGCAGATCTCGCTGTCTCAGTAACAAC
luc-1PRF-75-R	CATCTTCAATGGTGGCAGATCTTACCGCTGTCTCAGTAAC
luc-1PRF-78-R	CATCTTCAATGGTGGCAGATCTTTTTACCGCTGTCTCAG
luc-1PRF-PN1-R	CATCTTCAATGGTGGCAGATCTTTTTACGGCAGTCTCAG
luc-1PRF-PN2-F	CACCTCGAGGTGTTTAAACTGCTAGCCGCCAGCGGCTTGACCGGCAGTGGTCGC
luc-1PRF-PN2-R	GGCAGATCTTTTTACGGCAGTCTCAGTAACAACTAAGCCGCCGCGACCACTGCCGGTCA
luc-1PRF-PN3-F	CACCTCGAGGTGTTTAAACTGCTAGCCGCCAGCGGCTTGACGGCGAACGGTCGC
luc-1PRF-PN3-R	GGCAGATCTTTTTACCGCTGTCTCAGTAACAACTAAGCCGCCGCGACCGTTCGCCGTCA
luc-1PRF-PN4-R	GGCAGATCTTTTTAGGCGAACCTCAGTAACAACTAAGCCGCCGCGACCGTTCGCCGTCA
luc-1PRF-R	CATCTTCAATGGTGGCAGATCTTGTCTCAGTAACAACTAAGC
luc-1PRF-IFC-F1	GCAGTATTTCTACACCTCGAGGAAACTCCAGGGCCTGAC
luc-1PRF-IFC-R1	AGCAGATTTAGACACTGCTCCTTAGTCAGGCC
luc-1PRF-IFC-F2	GTGTCTAAATCTGCTAGCCGCCAGC
luc-1PRF-IFC-R2	ATCTTCAATGGTGGCAGATCTGAATTTGACTATTTTTACCGCTG
sgdluc-2prf-F	AACCCCGGGCCCTACTGCCAGGTTTTTAGCCTCG
sgdluc-ifc-F	AACCCCGGGCCCTACTGCCAGGTATTCTTAGCCTCGTTTCCCAT
sgdluc-2prf-R	CTTATGCCGTGCCTCTCGGAGAATAACCACTGTCAG
P1650-F	GAGCTCTTACGCGTGCTAGCTCACTAATAGAGCAGGCTCC
P1000-F	GAGCTCTTACGCGTGCTAGCCCACAAAGAACCATCCAGC
P500-F	GAGCTCTTACGCGTGCTAGCCACCGCCCCCGCCAC
P200-F	GAGCTCTTACGCGTGCTAGCTGACCGACTGCAGAGGCCG
P100-F	GAGCTCTTACGCGTGCTAGCGCAGAGGCGGAGCCAAGG
Promoter-R	AGTACCGGAATGCCAAGCTTCTCCAGGCGGGCTCCGCC
B500-R	AGTACCGGAATGCCAAGCTTTGTGTTTGCCAGGATTCGAT
B200-R	TACCGGAATGCCAAGCTTCATGTCCCCACGCTGCG
B100-R	AGTACCGGAATGCCAAGCTTTCTTGCGCCCCAAACCCAG

### CRISPR/Cas9 genome editing

MARC-145 cells with SHFL knockout (MARC-SHFL-KO) were generated using the CRISPR/Cas9 editing technology. Two guide RNAs (sgRNAs) targeting the monkey *shfl* gene were designed using the E-CRISP online tool (https://www.e-crisp.org) and cloned into the lentiCRISPR-dual vector. Lentiviral particles were generated in HEK-293T cells by cotransfecting the constructed lentiCRISPR-sgRNA plasmid with packaging plasmids psPAX2 and pCMV-VSV-G using Lipofectamine 2000 transfection reagent (Thermo Fisher) according to the manufacturer’s instructions. At 48 h post-transfection (hpt), culture supernatant was collected and centrifuged at 4°C, 4,000 rpm for 10 min. MARC-145 cells were transduced with the lentivirus supernatant supplemented with 8 μg/mL polybrene (YESAN Biotech) and selected with puromycin at 10 μg/mL to establish a polyclonal knockout pool.

The knockout efficiency was validated by treating MARC-SHFL-KO cells with IFN-α2b (0, 10, 100 IU/mL) for 12 h and analyzing endogenous SHFL expression via Western blot.

### Viral growth curve

To compare the growth kinetics of PRRSV in MARC-145 cells and MARC-SHFL-KO cells, one-step growth curve assays were performed. Briefly, MARC-145 and MARC-SHFL-KO cells seeded in 24-well plates were infected with rTA-EGFP at an MOI of 3. At 2 h post-infection (hpi), cells were washed three times with 1× PBS and maintained in MEM supplemented with 2% FBS. The culture supernatants were harvested at 8, 12, 16, 20, and 24 hpi, and virus titers were determined by the TCID_50_ assay.

### Attachment and internalization assay

MARC-145 cells at around 50% confluence in 12-well plates were transfected with a siRNA (Monkey-siSHFL, 70 pmol) against *shfl* or a control siRNA (siNC, 70 pmol) listed in Table 2. At 48 hpt, cells were pre-chilled at 4°C for 1 h before infection with rTA-EGFP (MOI = 3) at 4°C for 1 h. Unbound viral particles were removed by three washes with ice-cold PBS. Cells were harvested for total cellular RNA extraction. Virions attached to cells and SHFL expression level were quantified by RT-qPCR using primers/probes listed in Table 3. For the internalization assay, following the attachment phase, cells were shifted to 37°C for 2 h to allow viral entry. Non-internalized virions were removed by treatment with pronase (1 mg/mL) at 4°C for 1 h. Following total RNA extraction, RT-qPCR was performed to quantify internalized virions and SHFL expression level.

**TABLE 2:** siRNAs used for gene silencing in this study

Name	Sense (5′−3′)	Antisense (5′−3′)
Pig-siSHFL-1	GCAGAAAGAUGGACAGGAACUTT	AGUUCCUGUCCAUCUUUCUGCTT
Pig-siSHFL-2	GGAAAUGCAGGAAGCGCUACGTT	CGUAGCGCUUCCUGCAUUUCCTT
Pig-siSHFL-3	GCUCAGCUGAGGACUGCUACATT	UGUAGCAGUCCUCAGCUGAGCTT
Monkey-siSHFL	GACAGAAGCCAACCUAGGCAUGUT	GACUGUCUUCGGUUGGAUGCGUACAAA
siNC	UUCUCCGAACGUGUCACGUdTdT	ACGUGACACGUUCGGAGAAdTdT

**TABLE 3:** Primers and probes for RT-qPCR quantification

Gene name	Primer (5′−3′)	Probe
PRRSV-M-qF	CGGCAARTGATAACCACGC	FAM-GTCGTCCGGCGTCCCGG-BHQ
PRRSV-M-qR	TGCCACCCAACACGAGG	
PRRSV-nsp9-qF	CGGCGGCTTAGTTGTTACTG	
PRRSV-nsp9-qR	TCAACCTCACTGGCCACTTT	
shfl-qF1 (MARC-145)	ATGAGGAAATTCGGCAGCGA	FAM-AGCGCCCAACTCCCGTGTGG-BHQ
shfl-qR (MARC-145)	ATGTCCCGGTCCTGTTTCAT	
shfl-F (HEK-293T)	CGGTTCCGCTGCCCTGAGGC	
shfl-R (HEK-293T)	GCTGCCGAATTTCCTCATCA	
actb-qF	CCCTGGAGAAGAGCTACGAG	HEX-CGGTTCCGCTGCCCTGAGGC-BHQ
actb-qR (MARC-145)	CAGGAAGGAAGGTTGGAAGAG	
actb-qR1 (HEK-293T)	GGAAGGAAGGCTGGAAGAGT	

### Assembly and release assay

MARC-145 cells at around 50% confluence in 12-well plates were transfected with Monkey-siSHFL or siNC (70 pmol). After 48 hpt, cells were infected with rTA-EGFP (MOI = 3) at 37°C for 12 h. To quantify viral production, the intracellular and extracellular viral genomic RNA were extracted and measured by RT-qPCR using primers targeting the PRRSV nsp9-coding region. In parallel, virus titer in the supernatant was determined by TCID_50_ assay. The packaging efficiency was calculated as the percentage of the virion-associated genomic RNA in the total viral genomic RNA. The release efficiency was calculated as the percentage of extracellular virions in the total virus yield.

### RNA replication assay

MARC-145 cells at around 50% confluence in 24-well plates were transfected with Monkey-siSHFL or siNC (40 pmol). After 48 hpt, cells were infected with rTA-EGFP (MOI = 0.1) at 37°C for 24 h. Total cellular RNA was extracted and reverse-transcribed into cDNA. The accumulation of viral RNAs, including positive-strand genomic RNA (+gRNA) and subgenomic RNAs (+sgRNA) and negative-strand genomic RNA (−gRNA) and subgenomic RNAs (−sgRNA), was quantified by RT‒qPCR as described previously (45). The expression of viral RNA was normalized to the expression of the gene β-actin. The relative expression levels of both positive- and negative-sense subgenomic RNAs were calculated by normalizing their expression values to those of genomic RNA.

### HP-PRRSV replicon assay

HEK-293T and BHK-21 cells were seeded in 24-well plates and transfected with siRNA at approximately 60% and 50% confluence, respectively. HEK-293T cells were transfected with Monkey-siSHFL or siNC (5 pmol), while BHK-21 cells were transfected with Pig-siSHFL-1 or siNC (50 pmol). Twelve hours later, cells were transfected with 1 μg of PRRSV replicon plasmid using Lipofectamine 3000 transfection reagent (Thermo Fisher Scientific). At 24 hpt, culture supernatants were harvested for the GLuc assay, and cell lysates were collected for Western blot.

### Viral RNA translation assay

MARC-145 cells at around 50% confluence in 24-well plates were transfected with Monkey-siSHFL or siNC (40 pmol). At 48 hpt, cells were infected with rTA-NLuc (MOI = 3) expressing an nsp2-NLuc fusion protein. At the indicated times (2, 4, 6, 8, 12 hpi), cell lysates were harvested for the NLuc assay and Western blot.

### Ribosomal frameshifting assay

To assess the effect of SHFL overexpression on −1 PRF or −2 PRF, the PRF reporter plasmid or IFC (1 μg) was cotransfected with increasing amounts of FLAG-tagged SHFL plasmid (0, 0.125, 0.25, 0.5 μg) in HEK-293T and BHK-21 cells using Lipofectamine 2000 transfection reagent (Thermo Fisher Scientific). At 24 hpt, cell lysates were analyzed by dual-luciferase assay and Western blot. For knockdown experiments, HEK-293T cells at around 60% confluence in 24-well plates were transfected with Monkey-siSHFL or siNC (5 pmol). BHK-21 and PK-1 cells, at around 50% confluence, were transfected with Pig-siSHFL-1 or siNC (50 pmol and 5 pmol, respectively). Twelve hours later, cells were transfected with the PRF reporter or IFC plasmid (1 μg).

To identify the key domain of SHFL required for modulating the PRF, the PRF reporter plasmid (1 μg) was cotransfected with various SHFL mutant constructs (0.5 μg) into HEK-293T or BHK-21 cells using Lipofectamine 2000 transfection reagent (Thermo Fisher Scientific). Notably, for all assays specific to –2 PRF, FLAG-tagged nsp1β (0.2 μg) was included in the transfection to transactivate PRF (10). Cell lysates were collected at 24 hpt for dual-luciferase assay and Western blot. The frameshifting efficiency was calculated by normalizing the FLuc/RLuc ratio to that of the IFC.

### siRNA and siRNA transfection

The control siRNA (NC) and gene-specific siRNAs were obtained from SYNBIO or IDT, as shown in Table 2. Cells were transfected with siRNA oligos using Lipofectamine RNAiMAX (Thermo Fisher Scientific) according to the manufacturer’s instructions.

### TCID_50_ assay

Virus titers were determined by TCID_50_ assay in MARC-145 cells and calculated according to the Reed-Muench method as described previously (46).

### Luciferase assay

For the dual-luciferase assay, a Dual Luciferase Reporter Assay Kit (Vazyme Biotech, Nanjing, China) was employed to measure the activity of RLuc and FLuc in cell lysates according to the manufacturer’s instructions. The Gaussia luciferase (GLuc) assay was performed with coelenterazine h substrate (Maokang Biochem, Shanghai, China). The substrate was prepared by diluting the coelenterazine h stock to 20 μM with PBS supplemented with 5 mM NaCl, pH 7.2. To measure the Gaussia activity, mix 100 μL of 10 μM coelenterazine h with 20 μL of the sample in a white 96-well plate. The NanoLuc luciferase (NLuc) assay was performed with Furimazine substrate (Boluteng, Guangzhou, China). The substrate was prepared by diluting furimazine stock to 0.0005 mg/mL with PBS. To measure the Nano luciferase activity, mix 100 μL of Furimazine with 20 μL of the sample in a white 96-well plate. All luciferase assays were performed in a white plate and acquired photon counts for 10 s using a SuPerMax 1800 (Flash Spectrum Biological Technology, Shanghai, China) plate reader.

### Western blot analysis

For Western blot analysis, cells were harvested using Cell Lysis Buffer for Western and IP (Biosharp, Hefei, China). Following centrifugation at 12,000 × *g*, 10 min, 4°C, cell lysates were mixed with 5× SDS-PAGE loading buffer and denatured by heating at 95°C for 5 min or at 37°C for 30 min. The proteins were separated on a sodium dodecyl sulfate-polyacrylamide gel and transferred onto a nitrocellulose membrane (Millipore, USA) for subsequent immunoblotting. Membranes were blocked with 5% skim milk in 1× PBS for 1 h at room temperature and subsequently probed with primary antibodies overnight at 4°C. After five washes with 1× PBS supplemented with 0.05% Tween-20, the membrane was incubated with HRP-conjugated goat anti-rabbit IgG or goat anti-mouse IgG secondary antibodies for 1 h at room temperature. Membranes were analyzed using an ECL substrate (Biosharp, Hefei, China) with a Tanon 5200 Multi-imaging system.

### Quantitative reverse transcription PCR (RT-qPCR)

Total RNA was extracted from PRRSV-infected and mock-infected cells using the RNAprep Pure Cell/Bacteria Kit (TIANGEN Biotech, China) according to the manufacturer’s instructions. Viral genomic RNA and *shfl* mRNA were quantified by RT-qPCR using the HiScript II One Step qRT-PCR SYBR Green Kit or HiScript II One Step qRT-PCR Probe Kit (Vazyme Biotech, Nanjing, China), and β-actin served as an endogenous control. The primers and probes for RT-qPCR are listed in Table 3.

### Immunofluorescence assay (IFA)

MARC-145 cells or PAMs grown in glass-bottom dishes or cell culture plates were inoculated with PRRSV strains. At the indicated times post-infection, cells were fixed with ice-cold methanol for 20 min. After being blocked with 1% bovine serum albumin (BSA) in 1× PBS at room temperature for 1 h, cell monolayers were incubated with primary antibodies diluted in 1% BSA at 4°C overnight. After five washes with PBS, cells were incubated with secondary antibodies at 37°C for 1 h. Cell nuclei were stained with 4′,6-diamidino-2-phenylindole (DAPI, Solarbio) for 10 min at room temperature, and images were captured using inverted fluorescence microscopy (Olympus).

### Virus ultraviolet (UV) inactivation assay

For UV light inactivation, rTA-EGFP (MOI = 1) was exposed to 5 J/cm^2^ UV light for 30 min. Subsequently, MARC-145 cells in a 12-well plate were either mock-treated, infected with rTA-EGFP (MOI = 1), or infected with the UV-inactivated rTA-EGFP (MOI = 1) for 24 h.

### SHFL promoter activity assay

A series of truncated SHFL promoter constructs (1 μg) was cotransfected with pRL-TK-Luc (10 ng) in HEK-293T cells. At 24 hpt, cell lysates were collected and analyzed by dual-luciferase assay. To screen for PRRSV viral proteins that suppress SHFL promoter activity, the SHFL promoter construct (1 μg) was cotransfected with pRL-TK-Luc (10 ng) and individual plasmids expressing each PRRSV viral protein (500 ng) in HEK-293T cells. At 24 hpt, cell lysates were collected and analyzed by dual-luciferase assay.

### Nucleo-cytoplasmic fractionation assay

To determine the subcellular localization of nsp12 and N, nuclear and cytoplasmic proteins were extracted using a Nuclear and Cytoplasmic Protein Extraction Kit (Beyotime Biotechnology, China) according to the manufacturer’s instructions. Briefly, MARC-145 cells in a 6-well plate were mock-infected or infected with rTA-12 (MOI = 1). At 12 hpi, cells were collected for the isolation of nuclear and cytosolic proteins. Cytoplasmic (2.5% of total) and nuclear (10% of total) extracts were used for Western blot analysis of nsp12 and N using specific antibodies.

### Statistical analysis

Statistical analyses were performed with GraphPad Prism software 9.5.1. *P*-values were calculated using the two-tailed unpaired Student’s *t*-test.

## RESULTS

### SHFL overexpression inhibits the recovery of PRRSV

**Fig 1:**
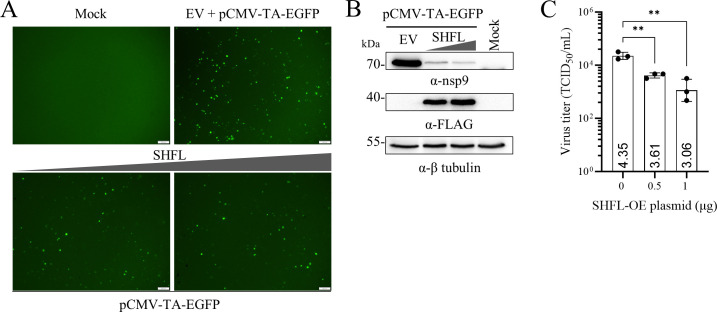
SHFL overexpression inhibits PRRSV infection. BHK-21 cells were cotransfected with a PRRSV infectious cDNA clone (pCMV-TA-EGFP, 2 μg) and FLAG-tagged SHFL plasmids (0, 0.5, or 1 μg). (**A**) The infection efficiency, reflected by the expression of the nsp2-EGFP fusion protein, was assessed via fluorescence microscopy at 48 hpt (scale bar, 100 μm). (**B**) The expression of nsp9 and FLAG-tagged SHFL in BHK-21 cells was evaluated by Western blot analysis. β-Tubulin was detected as a loading control. (**C**) Culture supernatants harvested from the transfected BHK-21 cells at 48 hpt were used to infect MARC-145 cells, and virus titers were determined by the TCID_50_ assay. Data are presented as means ± SD of three replicates. Statistical significance was analyzed using a two-tailed unpaired Student’s *t*-test. **, *P* < 0.01.

To evaluate the inhibitory effect of SHFL on PRRSV infection, BHK-21 cells were cotransfected with an infectious cDNA clone of the TA-12 strain (pCMV-TA-EGFP) and a plasmid expressing FLAG-tagged SHFL. SHFL overexpression led to a marked reduction in PRRSV infection, as evidenced by decreased expression of the nsp2-EGFP fusion protein and nsp9 in a dose-dependent manner (Fig. 1A and B). TCID_50_ assay with the culture supernatants revealed a 5.5- to 20-fold decrease in viral titers caused by the expression of SHFL (Fig. 1C), confirming that SHFL serves as a restriction factor against PRRSV infection.

### SHFL restricts PRRSV infection irrespective of species

**Fig 2:**
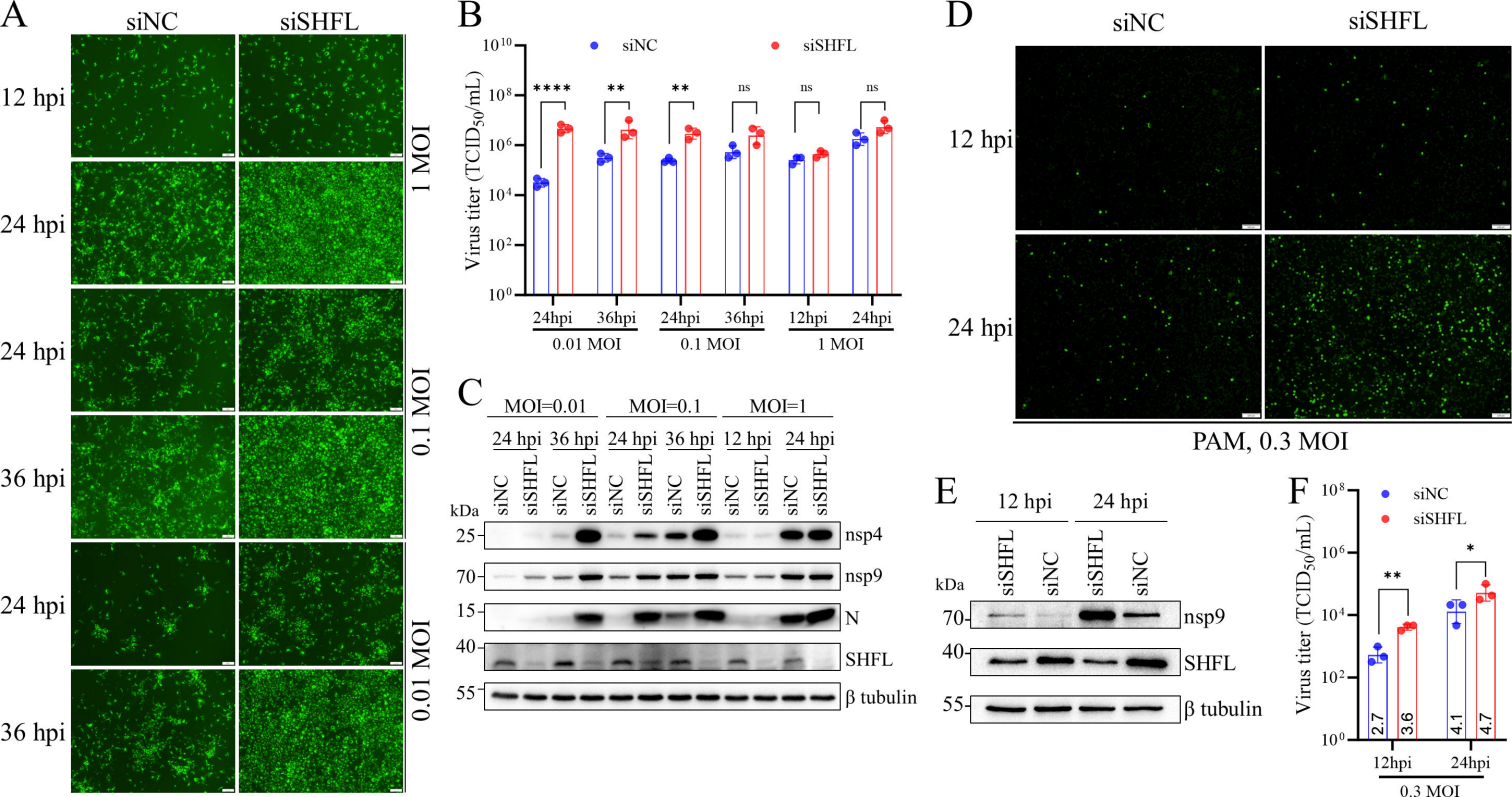
siRNA knockdown of SHFL enhances PRRSV infection in MARC-145 cells and PAMs. (**A–C**) MARC-145 cells were transfected with Monkey-siSHFL or siNC for 48 h. Cells were infected with rTA-EGFP (MOI = 0.01, 0.1, 1) and harvested at the indicated time points for Western blot analysis, and culture supernatants were harvested for virus titration. The replication kinetics of rTA-EGFP were monitored by EGFP signal under fluorescence microscopy (scale bar, 100 μm) (**A**). The viral titers in the supernatant were determined by the TCID_50_ assay in MARC-145 cells (**B**). The expression of viral proteins (nsp4, nsp9, and N) and endogenous SHFL was evaluated by Western blot analysis (**C**). (**D–F**) PAMs were transfected with siNC or siSHFL (Pig-siSHFL-1–3) for 48 h and then infected with NL1207 (MOI = 0.3). PRRSV replication was evaluated by IFA using a mAb against N protein (scale bar, 100 μm) (**D**), Western blot analysis of viral nsp9 (**E**), and virus yields in culture supernatants (**F**). Data presented in panels B and F are means ± SD of three replicates. Statistical significance was analyzed using multiple unpaired *t*-tests. *, *P* < 0.05; **, *P* < 0.01; ****, *P* < 0.0001; ns, not significant.

Next, we verified the antiviral effect of SHFL on PRRSV infection by a gene silencing experiment. As indicated by the EGFP signal, the infection of rTA-EGFP in MARC-145 cells with endogenous SHFL silenced was markedly enhanced compared to the negative control (siNC) (Fig. 2A). Viral yields in culture supernatants and viral protein expression were further determined by the TCID_50_ assay and Western blot analysis. SHFL knockdown enhanced the growth of rTA-EGFP at different infection doses, with a statistically significant difference observed when the infection doses were 0.01 and 0.1 MOI (Fig. 2B). Consistently, the expression levels of viral proteins (nsp4, nsp9, and N) were upregulated in cells with SHFL silenced (Fig. 2C). PAMs are the primary target cells for PRRSV replication *in vivo*, which could mimic the *in vivo* kinetics of PRRSV infection as much as possible. The antiviral effect of SHFL on PRRSV was further evaluated in PAMs using the NADC30-like NL1207 strain. SHFL expression in PAMs was knocked down by siRNA transfection. SHFL knockdown significantly increased viral replication at 12 and 24 hpi, as indicated by the increased expression of N protein (Fig. 2D). This enhancement was supported by increased levels of nsp9 (Fig. 2E) and approximately eightfold and fourfold increases in virus yield at 12 and 24 hpi (Fig. 2F). These results also suggest that endogenous SHFL exerts potent antiviral activity against PRRSV.

**Fig 3:**
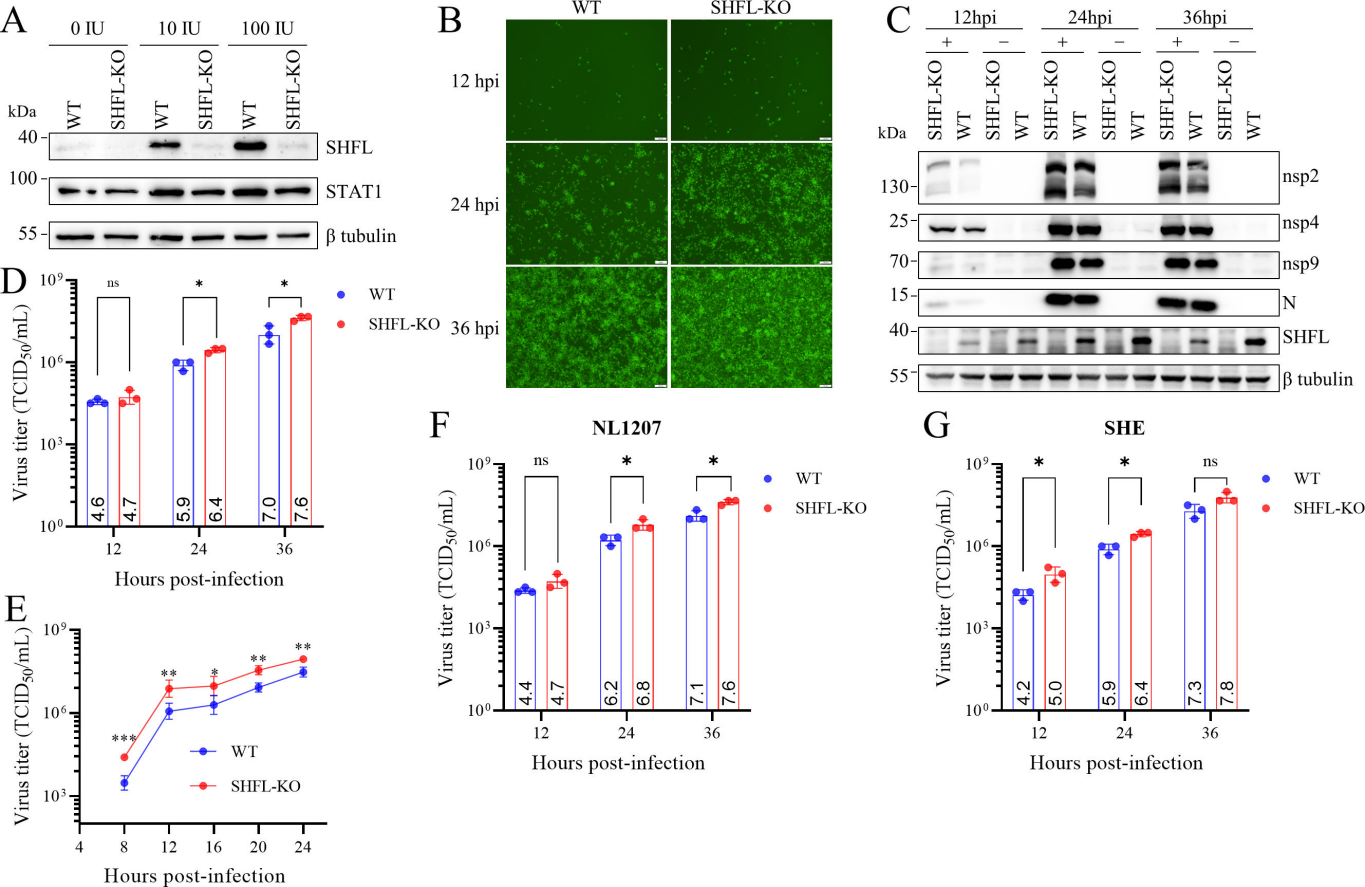
SHFL knockout enhances PRRSV infection irrespective of viral species. (**A**) Polyclonal MARC-145 cells with SHFL knockout by CRISPR/Cas9 gene editing. Endogenous SHFL expression in WT and SHFL-KO MARC-145 cells treated with IFN-α2b (0, 10, and 100 IU/mL) for 12 h was evaluated by Western blot analysis. (**B–D**) SHFL knockout enhanced the infection of rTA-EGFP. WT and SHFL-KO MARC-145 cells were infected with rTA-EGFP (MOI = 0.1). The infection of TA-EGFP in WT and SHFL-KO MARC-145 cells at the indicated time points was assessed by green fluorescence signal (B; scale bar, 100 μm), Western blot analysis of viral protein expression (**C**), and virus titers in culture supernatants (**D**). (**E**) One-step growth curves of rTA-EGFP in WT and SHFL-KO MARC-145 cells. Cells were infected with rTA-EGFP (MOI = 3). At the indicated times, supernatants were harvested and subjected to viral titration in MARC-145 cells. (**F and G**) SHFL knockout enhanced the infection of the NL1207 strain and the SHE strain. WT and SHFL-KO MARC-145 cells were inoculated with the NL1207 strain (MOI = 0.1) or the SHE strain (MOI = 0.1). At the indicated times, culture supernatants were harvested for virus titration. (**D–G**) Data presented are means ± SD from three replicates. Statistical significance was analyzed using multiple unpaired *t*-tests. *, *P* < 0.05; **, *P* < 0.01; ***, *P* < 0.001; ns, not significant.

To further validate the results generated by gene silencing, we generated a polyclonal MARC-145 cell strain with SHFL knockout using CRISPR/Cas9 gene editing technology. Since SHFL is an interferon-stimulated gene, we verified the loss of SHFL expression in cells treated with IFNα2b. As expected, we detected SHFL in MARC-145 (WT) cells but barely in the MARC-145-SHFL-KO (SHFL-KO) cells by Western blot analysis (Fig. 3A). We compared the infection of rTA-EGFP in SHFL-KO and WT cells. When the inoculation dose was 0.1 MOI, SHFL knockout boosted fluorescent signals (Fig. 3B), viral protein levels (Fig. 3C), and viral yields (Fig. 3D). One-step growth kinetics of rTA-EGFP in SHFL-KO and WT cells were also determined using an MOI of 3. In line with the results using the lower infection dose, viral replication in SHFL-KO cells was significantly enhanced compared to that in WT cells at all time points (Fig. 3E). Given the high genetic variability of PRRSV, it is important to define the breadth of SHFL’s antiviral activity against distinct strains. We therefore infected WT and SHFL-KO cells with the PRRSV-1 strain SHE and the NADC30-like PRRSV-2 strain NL1207. In line with earlier data, both strains produced significantly higher viral titers in SHFL-KO cells (Fig. 3F and G). Thus, SHFL exerts potent anti-PRRSV activity against all tested strains, indicating that its restrictive function is conserved across PRRSV species.

### SHFL restricts PRRSV infection by targeting the biosynthesis steps

**Fig 4:**
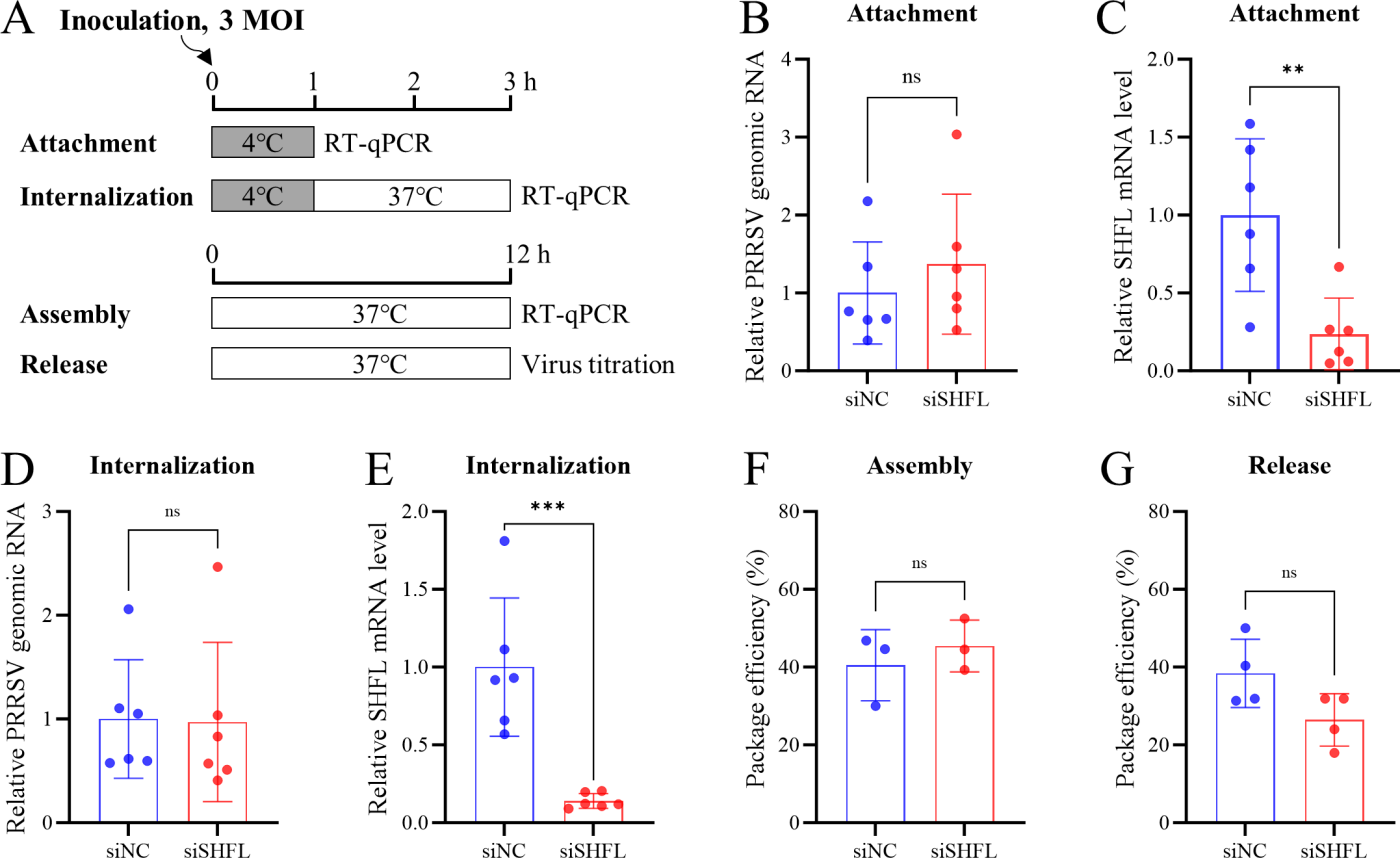
SHFL inhibits viral translation at the early stage of the PRRSV life cycle. (**A**) Schematic diagram of virus attachment, internalization, assembly, and release assays. (**B and C**) Effect of SHFL knockdown on the attachment of PRRSV. Cell-bound virions and SHFL expression levels were quantified by RT-qPCR. (**D and E**) Effect of SHFL knockdown on the internalization of PRRSV. Internalized virions and SHFL expression levels were quantified by RT-qPCR. (**F**) Effect of SHFL knockdown on the assembly of PRRSV. The packaging efficiency was calculated as the percentage of virion-associated genome in the total viral genome. (**G**) Effect of SHFL knockdown on the release of PRRSV. The release efficiency was calculated as the percentage of extracellular virions in the total virus yield. (**B–G**) Data are presented as means ± SD from three or six replicates. Statistical significance was analyzed using the two-tailed unpaired Student’s *t*-test. **, *P* < 0.01; ***, *P* < 0.001; ns, not significant.

**Fig 5:**
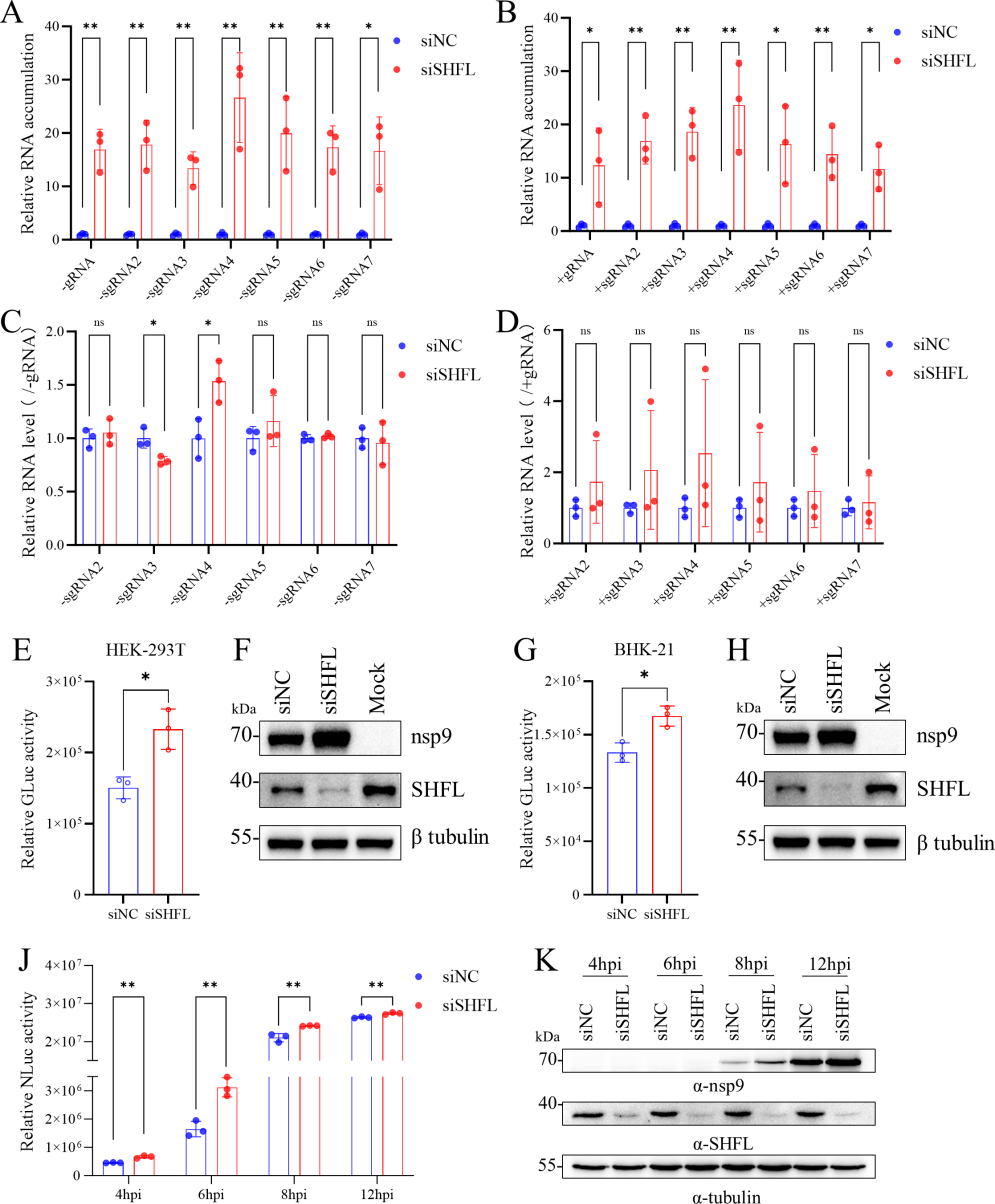
SHFL inhibits PRRSV RNA synthesis. (**A and B**) SHFL suppressed the synthesis of negative-sense and positive-sense viral RNAs. MARC-145 cells transfected with Monkey-siSHFL or siNC were infected with rTA-EGFP. Cells were harvested for total cellular RNA extraction. The accumulation of viral RNAs, including positive-strand genomic RNA (+gRNA) and subgenomic RNAs (+sgRNA) (**A**) and negative-strand genomic RNA (−gRNA) and subgenomic RNAs (−sgRNA) (**B**), was quantified by RT-qPCR as described previously (47). The expression of viral RNA was normalized to the expression of β-actin. (**C and D**) SHFL had no significant effect on the relative expression levels of both negative-sense (**C**) and positive-sense subgenomic RNAs (**D**), which were calculated by normalizing their expression values to those of genomic RNA. (**E–H**) The effect of SHFL on viral biosynthesis was evaluated using an HP-PRRSV replicon (47). The endogenous SHFL was knocked down in HEK-293T (Monkey-siSHFL, 5 pmol) and BHK-21 cells (Pig-siSHFL-1, 50 pmol). Twelve hours later, cells were transfected with the PRRSV replicon. Cell supernatants were collected at 24 hpt and analyzed by the GLuc assay (**E, G**). Cell lysates were subjected to Western blot analysis of nsp9 and endogenous SHFL. β-Tubulin was detected as a loading control (**F, H**). (**J and K**) SHFL suppressed viral translation at the early stage of PRRSV infection. MARC-145 cells transfected with Monkey-siSHFL or siNC were infected with rTA-NLuc (MOI = 3) expressing nsp2-NLuc. At the indicated times, cell lysates were harvested for the NLuc assay and Western blot analysis. (**A–J**) Data are presented as means ± SD from three replicates. Statistical significance was analyzed using the two-tailed unpaired Student’s *t*-test. *, *P* < 0.05; **, *P* < 0.01; ns, not significant.

To determine which stage of the PRRSV life cycle is affected by SHFL, we first evaluated the efficiency of viral attachment, entry, assembly, and release following SHFL knockdown (Fig. 4A). SHFL mRNA levels in knockdown cells were reduced to approximately 15% of those in negative control cells (Fig. 4C and E). Notably, SHFL knockdown did not significantly affect viral attachment (Fig. 4B), entry (Fig. 4D), assembly (Fig. 4F), or release (Fig. 4G). We next examined whether SHFL influences viral RNA synthesis using RT-qPCR assays as described previously (47). SHFL knockdown markedly increased the levels of both positive- and negative-sense genomic and subgenomic RNAs compared to the control (Fig. 5A and B). Given the importance of the optimal genomic-to-subgenomic RNA ratio for efficient viral RNA synthesis (48), we further quantified the relative expression of subgenomic RNAs normalized to genomic RNA. This analysis revealed only a slight, statistically insignificant increase in relative subgenomic RNA levels upon SHFL knockdown (Fig. 5C and D).

To further corroborate these findings, we employed a subgenomic replicon (47) to bypass the entry step and focus specifically on viral RNA translation and replication. SHFL knockdown significantly increased GLuc activity, a reporter for replicon RNA replication, in both HEK-293T and BHK-21 cells (Fig. 5E through G). The increase was more modest in BHK-21 cells, potentially due to their lower basal expression of endogenous SHFL and known interferon-deficient status. Consistently, Western blot analysis confirmed a pronounced increase in the level of nsp9 (Fig. 5F and H). As the replicon system does not exclude contributions from the translation step, we specifically assessed the impact of SHFL on PRRSV RNA translation using a rTA-NLuc reporter virus. In virus-infected cells, an nsp2-NLuc fusion protein is translated with viral genomic RNA immediately following its release in the cytoplasm. SHFL knockdown significantly enhanced NLuc activity during early infection, particularly at 4 and 6 hpi (Fig. 5J and K), indicating that SHFL inhibits the viral RNA translation step.

Collectively, these data demonstrate that SHFL suppresses PRRSV infection by specifically targeting viral RNA translation and replication, without obviously affecting attachment, entry, assembly, or release.

### Both the slippery sequence and downstream pseudoknot are required for canonical -1 PRF in PRRSV

**Fig 6:**
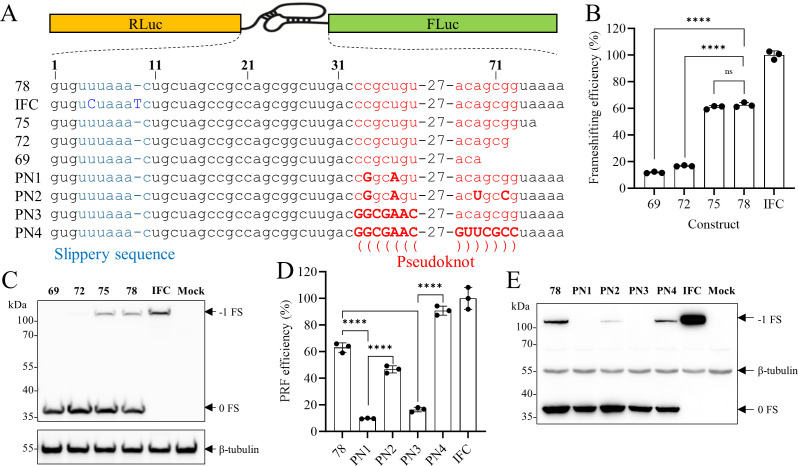
Mapping the RNA signal required for programmed −1 ribosomal frameshifting in PRRSV. (**A**) Schematic diagram of the dual-luciferase reporter carrying the putative −1 PRF signal or its mutants. (**B and C**) The minimal sequence required for efficient PRRSV –1 PRF is 75 nt. Truncated mutants of –1 PRF or IFC were transfected into HEK-293T cells. At 24 hpt, cell lysates were harvested for the dual-luciferase assay (**B**) and Western blot analysis (**C**). (**D and E**) Mutational analysis of the predicted pseudoknot structure. The WT plasmid, pseudoknot mutants, or IFC were transfected into HEK-293T cells. At 24 hpt, cell lysates were analyzed by dual-luciferase assay (**D**) and Western blot analysis (**E**). The expression of the pDluc translation products was detected by an antibody recognizing Renilla luciferase. β-Tubulin was detected as a loading control. Data are presented as means ± SD from three replicates. Statistical significance was analyzed using the two-tailed unpaired Student’s *t*-test. ****, *P* < 0.0001; ns, not significant.

Given the essential role of −1 PRF in synthesizing viral RTC for RNA replication (1), together with our finding that SHFL suppresses viral RNA replication, we hypothesized that SHFL targets −1 PRF to modulate this process. To test this, we first defined the core −1 PRF RNA signal of PRRSV using a dual-luciferase reporter system. A sequence spanning the ORF1a/1b overlapping region of the PRRSV-2 TA-12 strain was inserted between an RLuc gene and a FLuc gene (placed in the −1 frame) (10). A series of 3′-end truncation mutants (each shortened by 3 nucleotides) was generated (Fig. 6A). Frameshift efficiency for each construct was calculated by normalizing the FLuc/RLuc ratio to that of an IFC construct. We determined that sequences of 75 nt and 78 nt were sufficient to induce efficient −1 PRF, yielding an efficiency of approximately 60% (Fig. 6B). Consistent with the luciferase activity data, a frameshift product with the expected molecular mass of ∼100 kDa was abundantly expressed in cells transfected with pDluc-1PRF-75 and pDluc-1PRF-78 (Fig. 6C). Thus, the minimal PRRSV −1 PRF signal requires 75 nt. Mfold analysis (http://www.unafold.org) predicted the presence of a probable RNA pseudoknot in this region. Of note, truncation to 72 nt and 69 nt, leading to the disruption of the predicted RNA pseudoknot, caused a sharp decline in frameshift efficiency (Fig. 6B). To validate this structural model, we designed mutants that partially (PN1) or completely (PN3) disrupted base-pairing of the predicted pseudoknot, along with corresponding compensatory mutants that restored pairing (PN2, PN4). Disruptive mutations severely impaired frameshifting, whereas compensatory mutations not only rescued but even enhanced efficiency to 40% and up to 80% (Fig. 6D and E), confirming that an RNA pseudoknot is required for efficient −1 PRF. We next compared the nucleotide sequences encompassing the −1 PRF signal across all available PRRSV strains. Alignment revealed that both the frameshift site and the pseudoknot are highly conserved, displaying nearly 100% sequence identity among strains (Fig. 1). In summary, these results demonstrate that the PRRSV −1 PRF signal is highly conserved across all strains and consists of a slippery sequence followed by a structurally essential downstream pseudoknot, both of which are required for efficient ribosomal frameshifting.

### Critical contribution of SHFL to the inhibition of −1 PRF

**Fig 7:**
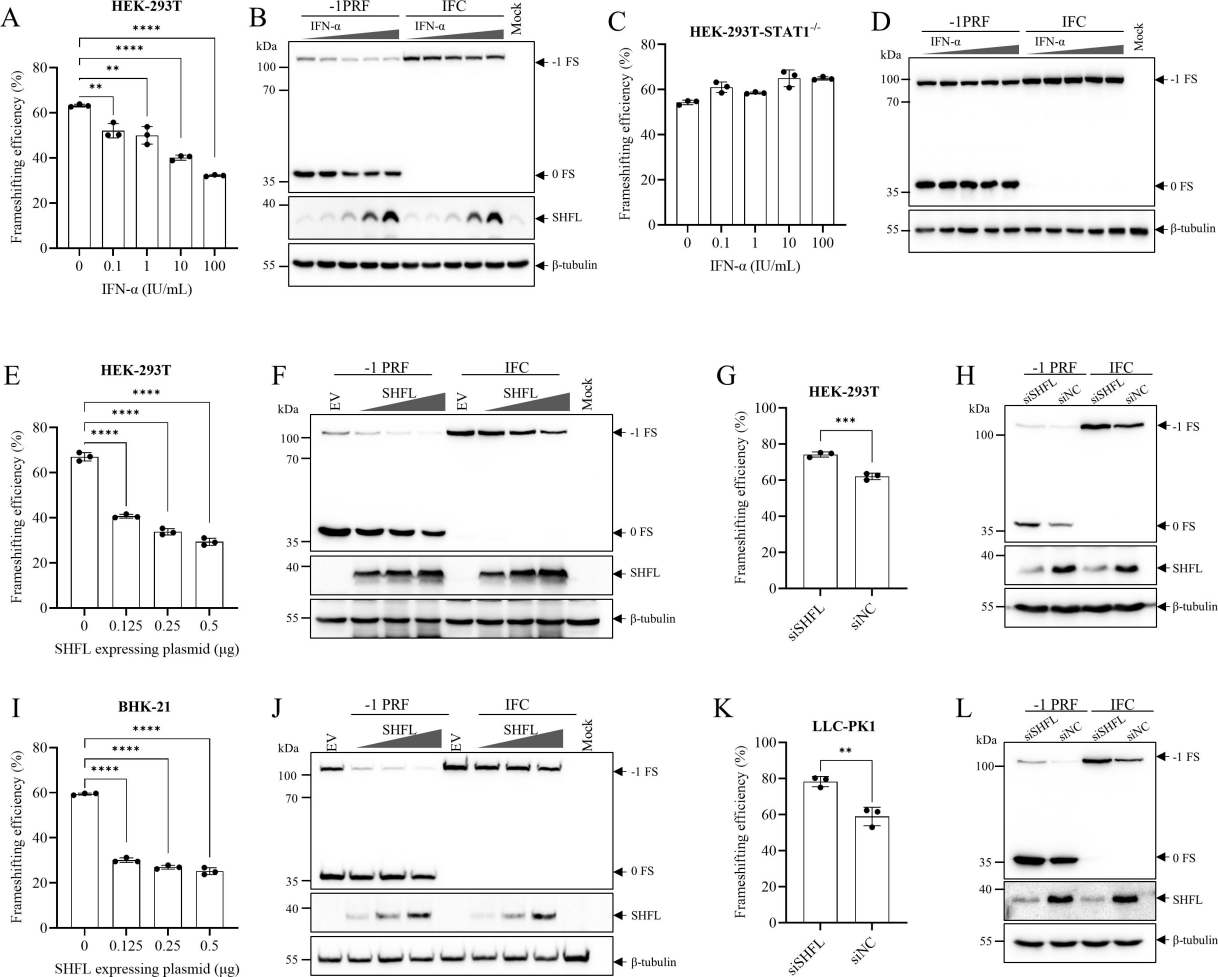
SHFL suppresses PRRSV −1 PRF. (**A–D**) ISG mediated the inhibitory effect of IFN on PRRSV −1 PRF. After pretreatment with 10-fold serially diluted IFN-α2b for 12 h, the −1 PRF reporter plasmid or IFC was transfected into HEK-293T (**A and B**) and STAT1-knockout HEK-293T cells (**C and D**). At 24 hpt, cell lysates were analyzed by dual-luciferase assay and Western blot analysis. (**E, F, I, J**) SHFL overexpression suppressed PRRSV −1 PRF in a dose-dependent manner. The −1 PRF reporter plasmid was cotransfected with increasing amounts of FLAG-tagged SHFL plasmid in HEK-293T (**E and F**) and BHK-21 (**I and J**) cells. At 24 hpt, cell lysates were analyzed by dual-luciferase assay and Western blot. (**G, H, K, L**) Knockdown of endogenous SHFL enhanced PRRSV −1 PRF. HEK-293T (**G and H**) and PK1 (**K and L**) cells were first transfected with siSHFL or siNC. Twelve hours later, the −1 PRF reporter plasmid or IFC was introduced. Cell lysates were collected at 24 hpt for dual-luciferase assays and Western blot using an anti-FLAG antibody to detect FLAG-tagged SHFL, an anti-RyDEN antibody to detect endogenous SHFL expression, and an anti-Renilla antibody to detect –1 PRF products. β-Tubulin was detected as a loading control. Data are presented as means ± SD from three replicates. Statistical significance was analyzed using the two-tailed unpaired Student’s *t*-test. **, *P* < 0.01; ***, *P* < 0.001; ****, *P* < 0.0001; ns, not significant.

Since SHFL is an interferon-stimulated gene, we initially tested whether PRRSV −1 PRF is sensitive to IFNα treatment. HEK-293T cells were pretreated with increasing doses of IFNα2b for 12 h before transfection with the −1 PRF reporter plasmid. IFNα2b treatment suppressed −1 PRF efficiency in a dose-dependent manner (Fig. 7A and B). Notably, this suppression was completely abrogated in STAT1-knockout HEK-293T cells, which are defective in IFN signaling and ISG induction (Fig. 7C and D). These results indicate that IFN-mediated inhibition of PRRSV −1 PRF requires ISG induction. We next examined whether SHFL specifically contributes to this inhibition. Overexpression of SHFL in HEK-293T (Fig. 7E and F) and BHK-21 cells (Fig. 7I and J) significantly inhibited PRRSV −1 PRF in a dose-dependent manner. Conversely, SHFL knockdown increased the efficiency of PRRSV −1 PRF by approximately 20% in both HEK-293T (Fig. 7G and H) and porcine kidney (PK1) cells (Fig. 7K and L). Together, these findings establish SHFL as a potent inhibitor of PRRSV −1 PRF.

**Fig 8:**
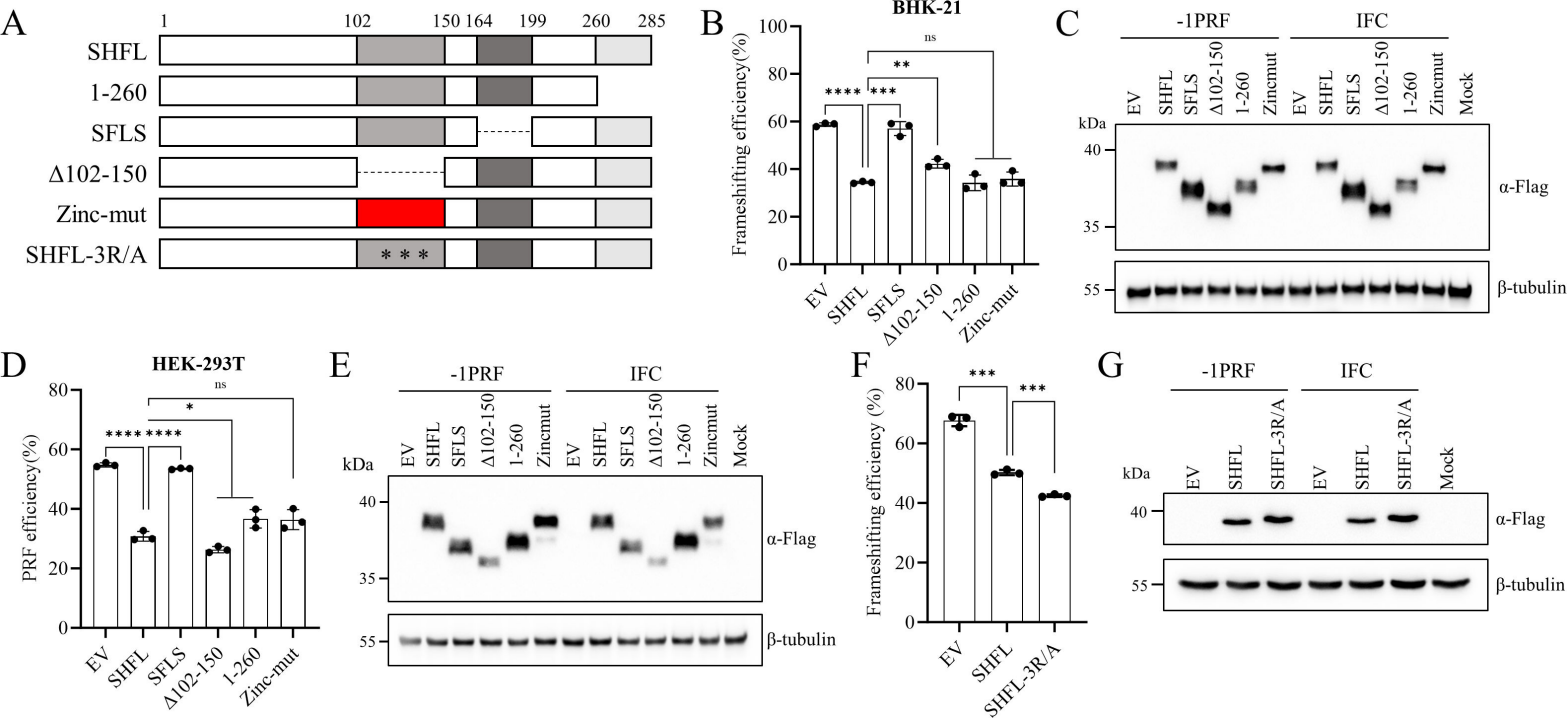
Identification of the key region essential for SHFL’s inhibitory effect on PRRSV −1 PRF. (**A**) Schematic diagram of SHFL domains and mutants. Deleted regions are represented by dashed lines; Mutation regions are highlighted in red; Key point mutations are marked by asterisks (*); Δ indicates the lack of the denoted region. (**B–G**) The 164th–199th amino acid region of SHFL is a key region involved in suppressing PRRSV −1 PRF. The −1 PRF reporter plasmid was cotransfected with different SHFL mutants in BHK-21 (**B and C**) and HEK-293T (**D and E**) cells. (**F and G**) The −1 PRF reporter plasmid was cotransfected with SHFL-3R/A mutant in HEK-293T cells. In all experiments, cell lysates were harvested at 24 hpt and analyzed by dual-luciferase assay and Western blot using an anti-FLAG antibody to detect SHFL mutant expression. β-Tubulin was detected as a loading control. (B, D, and F) Data are presented as means ± SD from three replicates. Statistical significance was analyzed using the two-tailed unpaired Student’s *t*-test. *, *P* < 0.05; **, *P* < 0.01; ***, *P* < 0.001; ****, *P* < 0.0001; ns, not significant.

To map the functional domains of SHFL responsible for this activity, we generated a series of SHFL mutants based on its structure (Fig. 8A). These constructs were transit-expressed in BHK-21 (Fig. 8B and C) and HEK-293T cells (Fig. 8D and E), with expression confirmed by Western blotting (Fig. 8C and E). Luciferase assays revealed that deletion of the 164th–199th amino acid region abolished SHFL’s ability to suppress frameshifting, while mutations in other regions retained inhibitory activity (Fig. 8B and D). Furthermore, mutation of three previously identified key RNA-binding sites within SHFL (44) did not impair its inhibitory function against PRRSV −1 PRF (Fig. 8F and G).

In summary, these data demonstrate that SHFL is a critical ISG responsible for inhibiting PRRSV −1 PRF, and that its functional activity depends on the integrity of the 164th–199th amino acid region.

### SHFL exerts an inhibitory effect on PRRSV −2 PRF

**Fig 9:**
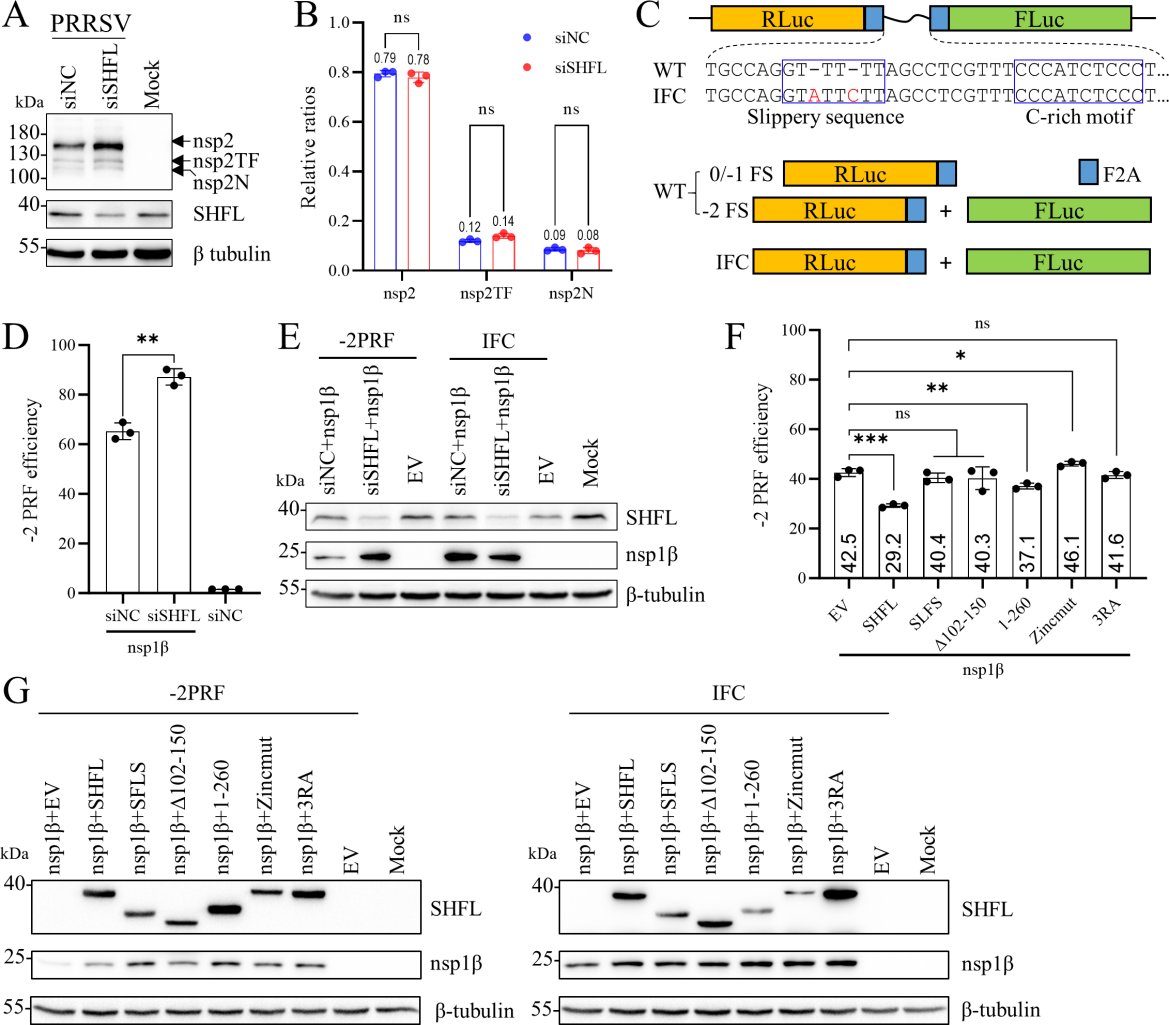
SHFL suppresses the –2 PRF of PRRSV. (**A and B**) SHFL had no significant effect on the novel –2/−1 PRF during PRRSV infection. MARC-145 cells were transfected with Monkey-siSHFL or siNC (70 pmol) for 48 h. Subsequently, cells were infected with rTA-EGFP (MOI = 1). Cell lysates were collected at 24 hpi for Western blot using an anti-nsp2 antibody to detect the expression of nsp2, nsp2TF, and nsp2N (**A**). Densitometric analysis of the relative ratios of nsp2, nsp2TF, and nsp2N (**B**). Data are presented as means ± SD from three replicates. Statistical significance was analyzed using the two-tailed unpaired Student’s *t*-test. ns, not significant. (**C**) Schematic diagram of the dual-luciferase reporter carrying –2/−1 PRF signal from PRRSV (WT) of IFC. (**D and E**) Knockdown of endogenous SHFL stimulated the −2 PRF of PRRSV. HEK-293T cells were first transfected with Monkey-siSHFL or siNC. Twelve hours later, the WT or IFC plasmid and FLAG-tagged nsp1β were introduced. Cell lysates were collected at 24 hpt for dual-luciferase assays (**D**) and Western blot (**E**) using an anti-FLAG antibody to detect nsp1β and an anti-RyDEN antibody to detect endogenous SHFL expression. (**F and G**) The effect of SHFL and its mutants on the −2 PRF of PRRSV. The WT or IFC plasmid and FLAG-tagged nsp1β plasmid were cotransfected with SHFL or its mutant plasmids in HEK-293T cells. Cell lysates were harvested at 24 hpt and analyzed by dual-luciferase assay (**F**) and Western blot (**G**) using an anti-FLAG antibody to detect nsp1β and SHFL mutant expression. β-Tubulin was detected as a loading control. Data are presented as means ± SD from three replicates. Statistical significance was analyzed using the two-tailed unpaired Student’s *t*-test. *, *P* < 0.05; **, *P* < 0.01; ***, *P* < 0.001; ns, not significant.

Although SHFL was originally characterized as a specific and broad-spectrum inhibitor of −1 PRF (30), recent studies indicate that it can also influence +1 PRF efficiency and modulate termination codon readthrough (44). This broader activity prompted us to hypothesize that SHFL might regulate the −2/−1 PRF utilized by PRRSV to translate nsp2TF and nsp2N. To quantify the efficiency of −2/−1 PRF during viral infection, we first analyzed the band intensity of nsp2-related proteins by Western blot analysis (Fig. 9A). This analysis revealed that −2/−1 PRF efficiency was not significantly altered upon SHFL knockdown (Fig. 9B). We subsequently employed a dual-luciferase reporter assay for a more sensitive quantification as described previously (43) (Fig. 9C). The reporter plasmid contained the −2/−1 PRF signal sequence of the TA-12 strain inserted between the RLuc and FLuc ORFs. Using this system, we found that SHFL knockdown increased −2 PRF efficiency from approximately 60% to 80% in HEK-293T cells (Fig. 9D and E), whereas SHFL overexpression decreased it (Fig. 9F). This confirms that SHFL suppresses PRRSV −2 PRF. To identify the domain of SHFL mediating this effect, we cotransfected the −2 PRF reporter with a series of SHFL truncation and point mutants. Interestingly, only full-length SHFL and 1–260 mutant inhibited −2 PRF (Fig. 9F and G). These results indicate that the structural integrity of SHFL is essential for its activity against −2 PRF, suggesting a mechanism distinct from its inhibition of canonical −1 PRF.

**Fig 10:**
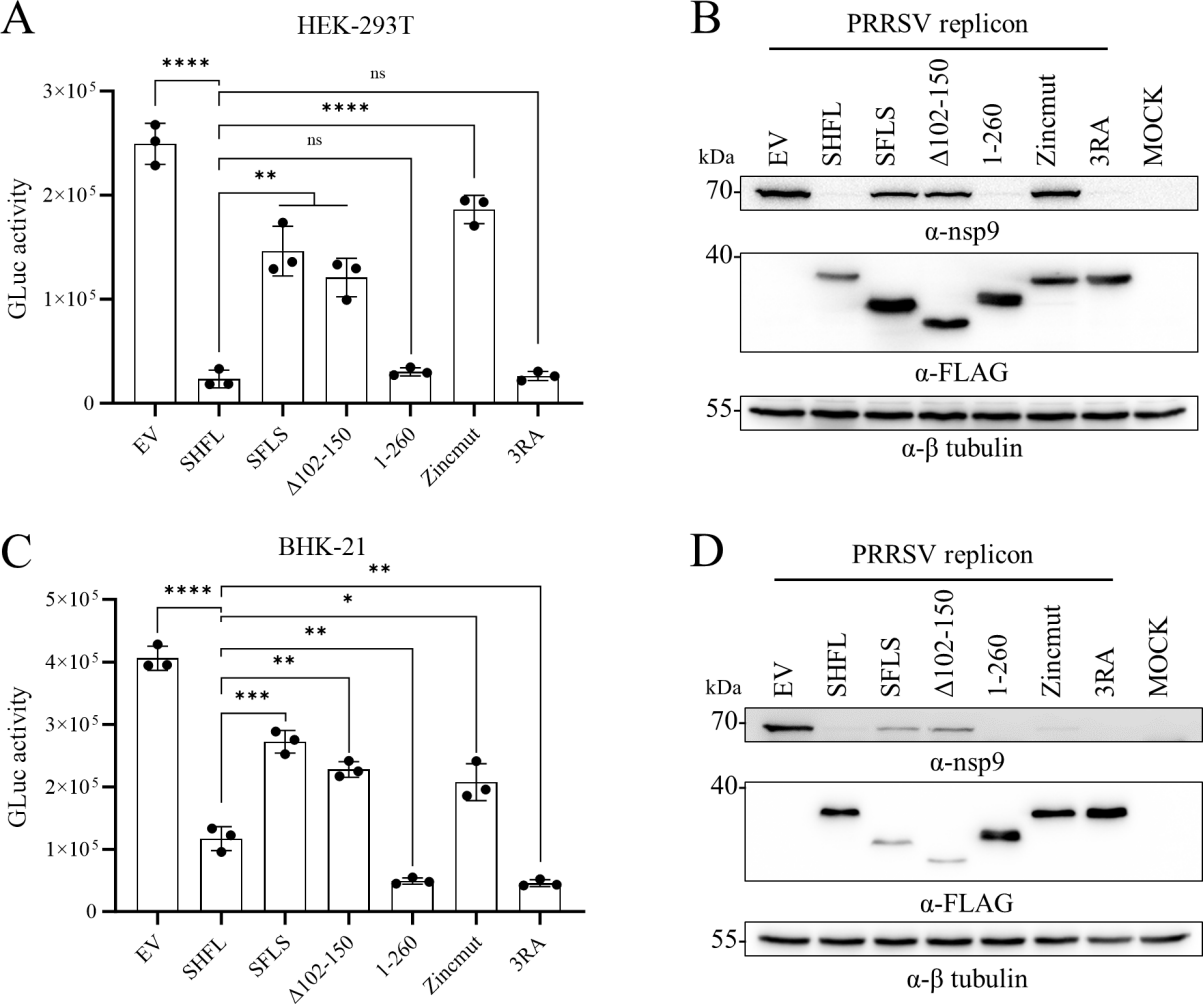
Identification of the key region essential for SHFL antiviral inhibitory effect on PRRSV. PRRSV replicon (1 μg) was cotransfected with SHFL or its mutant plasmids (0.5 μg) in HEK-293T and BHK-21 cells. At 24 hpt, cell supernatants were harvested for the GLuc assay (**A and C**) and cell lysates for Western blot (**B and D**) using an anti-FLAG antibody to detect SHFL mutant expression and an anti-nsp9 antibody to detect RNA replication. β-Tubulin was detected as a loading control. Data are presented as means ± SD from three replicates. Statistical significance was analyzed using the two-tailed unpaired Student’s *t*-test. *, *P* < 0.05; **, *P* < 0.01; ***, *P* < 0.001; ****, *P* < 0.0001; ns, not significant.

To further examine whether SHFL mainly targets PRF events to inhibit PRRSV replication, we co-transfected the PRRSV replicon with SHFL mutants in HEK-293T and BHK-21 cells. The WT SHFL and all mutants inhibited replicon activity to varying degrees (Fig. 10A and C), although SFLS was unable to suppress −1 PRF (Fig. 8D) and −2 PRF (Fig. 9F). In comparison with WT SHFL, three mutants (SFLS, ∆102–150, and Zincmut) exhibited a significantly attenuated inhibitory effect on viral replicon. These results confirm that SHFL employs additional anti-PRRSV mechanisms beyond PRF inhibition, such as suppressing viral mRNA translation.

### PRRSV nsp12 and N downregulate SHFL expression at the transcriptional level

**Fig 11:**
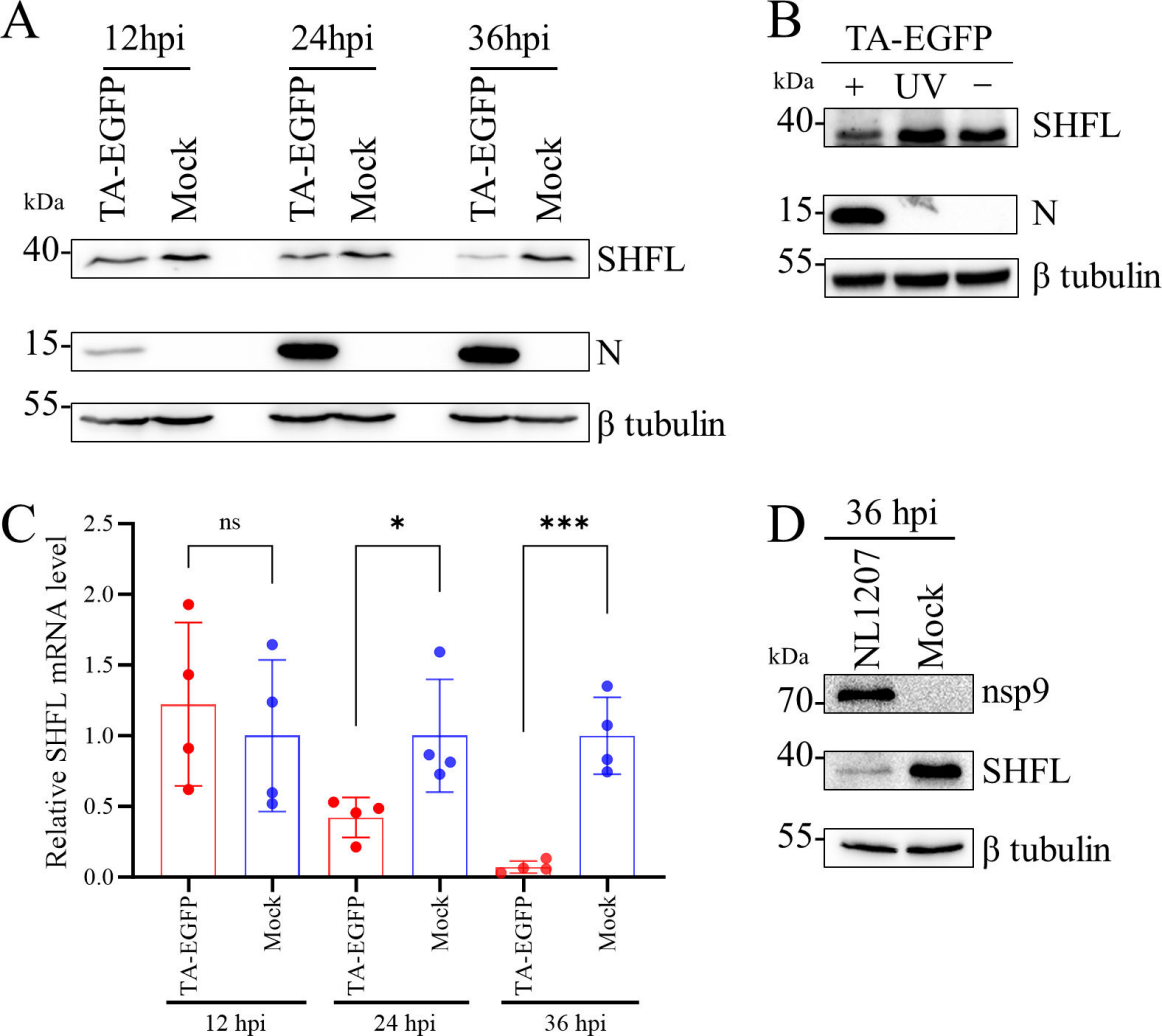
PRRSV infection downregulates SHFL expression transcriptionally. (**A**) PRRSV infection downregulates SHFL expression. MARC-145 cells were either infected with rTA-EGFP (MOI = 0.1) or mock-infected. Cell lysates at the indicated times were harvested for Western blot. (**B**) SHFL expression in MARC-145 cells that were mock-treated, infected with rTA-EGFP (MOI = 1), or UV-inactivated rTA-EGFP (MOI = 1) for 24 h. Cell lysates were harvested for Western blot. (**C**) Relative SHFL mRNA levels in mock-treated and rTA-EGFP-infected (MOI = 0.1) MARC-145 cells at the indicated times. The mRNA levels were normalized to β-actin as determined by RT-qPCR. Data are presented as means ± SD from four replicates. Statistical significance was analyzed using the two-tailed unpaired Student’s *t*-test. *, *P* < 0.05; ***, *P* < 0.001; ns, not significant. (**D**) The downregulation of SHFL expression existed in PAMs during PRRSV infection. PAMs were either infected with the NL1207 strain (MOI = 0.3) or mock-infected. Cell lysates at the indicated times were harvested for Western blot. β-Tubulin was detected as a loading control.

Previous results indicated that endogenous SHFL expression is suppressed in MARC-145 cells infected with rTA-EGFP across various MOI (Fig. 2C and 3C). As SHFL is an interferon-stimulated gene typically upregulated during viral infection, its downregulation here suggests that PRRSV may downregulate its expression to evade the antiviral effect. To substantiate this hypothesis, we first confirmed that PRRSV infection indeed reduces SHFL expression in PRRSV-infected cells (Fig. 11A). Moreover, SHFL suppression was observed only with replication-competent virus, not with UV-inactivated PRRSV, indicating the requirement for active viral replication (Fig. 11B). Notably, SHFL mRNA levels were also lower in infected MARC-145 cells compared to uninfected controls (Fig. 11C), suggesting that PRRSV interferes with SHFL transcription. A similar downregulation of SHFL protein was verified in PRRSV-infected PAMs by Western blot analysis (Fig. 11D).

**Fig 12:**
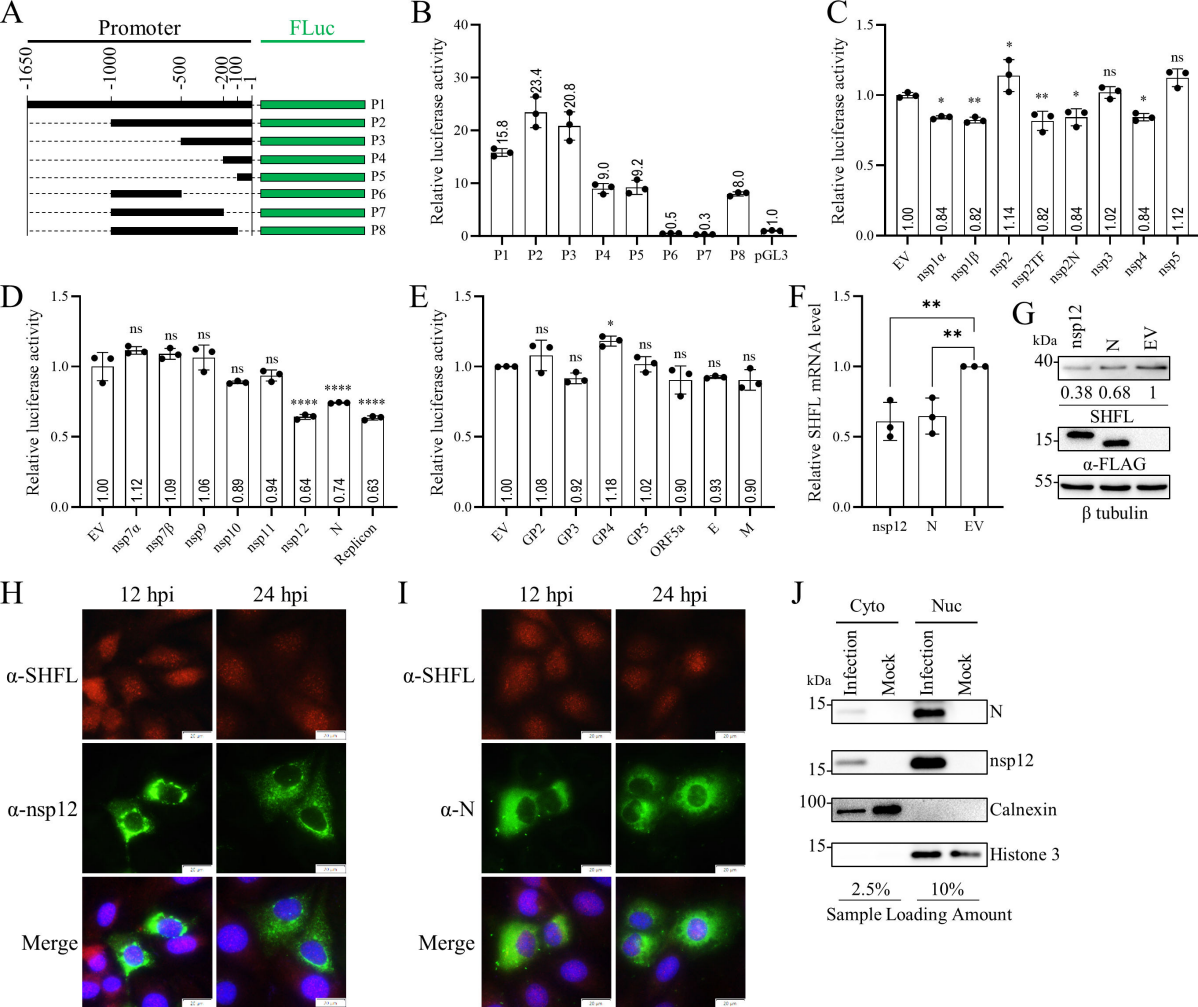
PRRSV nsp12 and N proteins inhibit SHFL transcription. (**A**) Schematic diagram of truncated SHFL promoter constructs (P1–P8). (**B**) Mapping the core promoter region of the pig *shfl* gene. A series of truncated SHFL promoter constructs (P1–P8) was cotransfected with the pRL-TK-Luc in HEK-293T cells. At 24 hpt, cell lysates were collected and analyzed by the dual-luciferase activity. (**C–E**) Screening for PRRSV proteins that suppress SHFL promoter activity. SHFL promoter construct (P2) was cotransfected with pRL-TK-Luc and individual plasmids expressing each PRRSV viral protein in HEK-293T cells. At 24 hpt, cell lysates were collected and analyzed by the dual-luciferase activity. (**F and G**) PRRSV nsp12 and N proteins inhibit SHFL transcription. HEK-293T cells were transfected with FLAG-tagged nsp12 or N (1 μg) for 36 h. Cells were harvested for total cellular RNA extraction, and endogenous SHFL mRNA levels were detected by RT-qPCR (**F**). Cell lysates were collected, and endogenous SHFL protein expression levels were analyzed by Western blot. β-Tubulin was detected as a loading control. Grayscales of SHFL/β-tubulin are shown below the figure panel G. (**C–F**) Statistical significance between empty vector (EV) and viral protein was analyzed using the two-tailed unpaired Student’s *t*-test. *, *P* < 0.05; **, *P* < 0.01; ****, *P* < 0.0001; ns, not significant. (**H and I**) The subcellular localization of nsp12 and N. MARC-145 cells in 35 mm dishes were mock-infected or infected with TA-12 (MOI = 1) for 12 h and 24 h, after which cells were fixed and processed for IFA using a mAb against nsp12 or N protein (scale bar, 20 μm). (**J**) The subcellular localization of PRRSV nsp12 and N in virus-infected MARC-145 cells was analyzed by nuclear and cytoplasmic fractionation.

Given the transcriptional downregulation of SHFL, we hypothesized that one or more PRRSV proteins might directly target the SHFL promoter or the associated transcription factors. We cloned a 1,650-bp fragment upstream of the *shfl* transcript from PK1 cells and generated a series of truncations into the pGL3-Basic luciferase vector (Fig. 12A). Dual-luciferase assays identified that at least 1,000 bp of the promoter were required for maximal activity (Fig. 12B). To identify viral proteins that modulate this promoter, we co-transfected HEK-293T cells with individual PRRSV protein-expressing plasmids along with the SHFL promoter reporter. Among all viral proteins tested, nsp12 and N significantly suppressed promoter activity, while nsp1α, nsp1β, nsp2TF, nsp2N, and nsp4 showed milder inhibitory effects (Fig. 12C through E). Overexpression of FLAG-tagged nsp12 or N significantly reduced SHFL mRNA and protein levels, as shown by RT-qPCR and Western blot analysis, respectively (Fig. 12F and G). Although the N protein is known to localize to both the cytoplasm and nucleolus, with nuclear import occurring rapidly (49), the nuclear localization of nsp12 was unclear. Confocal microscopy revealed that both nsp12 and N were present in the cytoplasm and nucleus of infected cells (Fig. 12H and I). Subcellular fractionation confirmed abundant nsp12 and N in both cytosolic and nuclear fractions, with minimal cross-contamination as indicated by the absence of calnexin in the nuclear fraction and histone H3 in the cytosolic fraction (Fig. 12J). Together, these results demonstrate that PRRSV nsp12 and N downregulate SHFL expression at the transcriptional level, likely through direct or indirect modulation of the SHFL promoter in the nucleus.

## DISCUSSION

**Fig 13:**
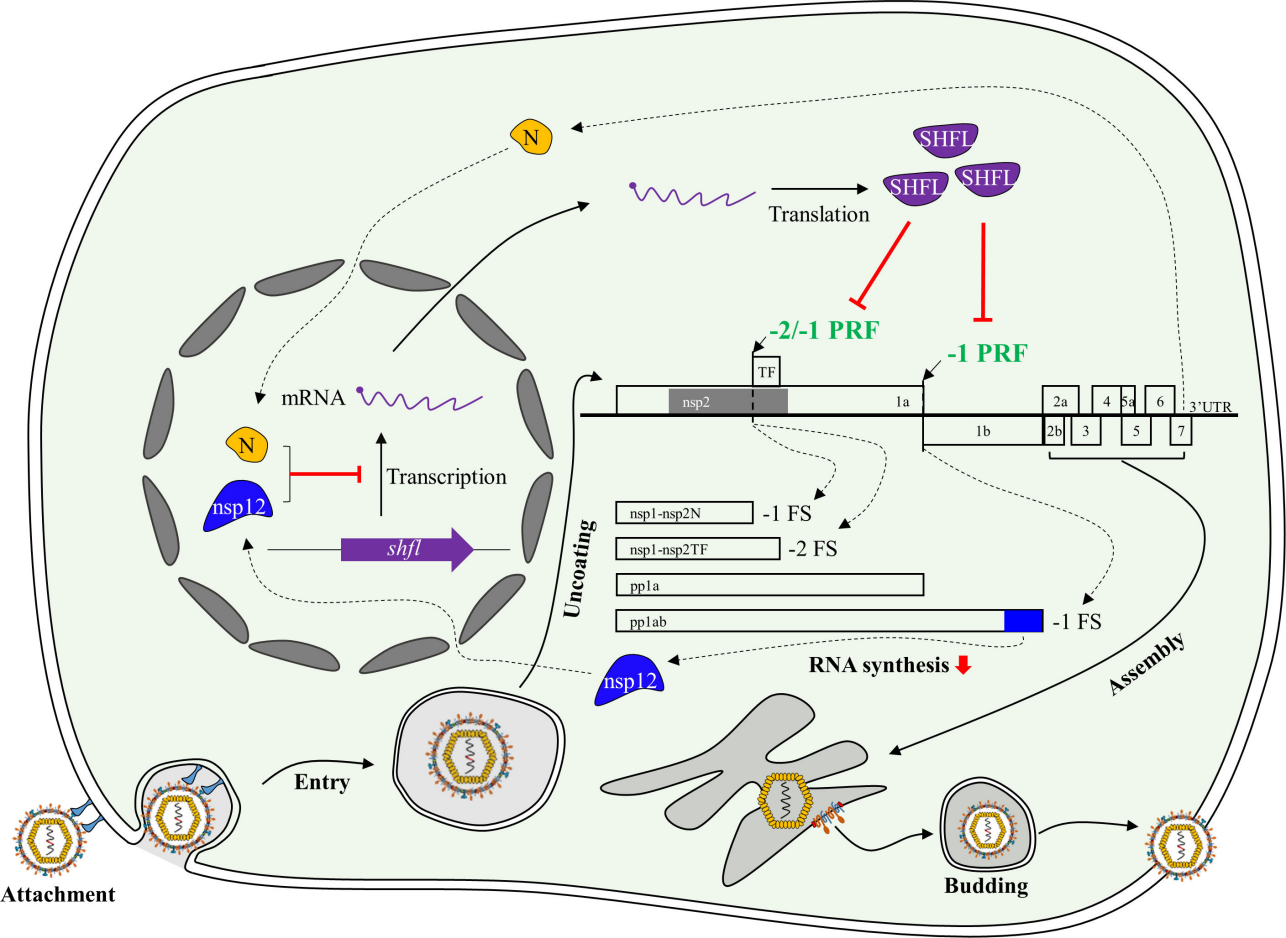
Overview of the interaction between SHFL and PRRSV infection. SHFL exerts its antiviral effect by specifically disrupting viral RNA translation and replication, without affecting attachment, entry, assembly, or release. Mechanistically, SHFL inhibits the essential −1 PRF of PRRSV. Since −1 PRF is required for generating the RTC components from ORF1b, its inhibition leads to impaired RNA synthesis. SHFL also suppresses PRRSV’s novel −2 PRF. In response, PRRSV deploys nsp12 and N proteins to antagonize SHFL’s antiviral activity transcriptionally.

PRRSV has been a major economically significant pathogen in the global swine industry for decades. Its high genetic variability poses a substantial challenge to the development of effective antiviral strategies. Therefore, identifying broad-spectrum inhibitors and elucidating the host innate immune responses that restrict PRRSV replication are urgently needed. In this study, we demonstrate that SHFL inhibits infection by both PRRSV species. As illustrated in Fig. 13, SHFL specifically blocks PRRSV RNA translation and replication by suppressing viral programmed ribosomal frameshifting events. Conversely, multiple viral proteins, especially nsp12 and N, are employed to evade this antiviral effect by downregulation of SHFL expression at the transcriptional level.

PRRSV belongs to the order *Nidovirales*, all members of which share a similar viral life cycle. A hallmark of nidovirus infection is the production of a 3′-coterminal nested set of subgenomic mRNAs, a process mediated by the RTC. The RTC consists of replicase subunits derived from polyproteins pp1a and pp1ab, along with certain host factors. The synthesis of pp1ab itself depends critically on a −1 PRF event within the ORF1a/ORF1b overlapping region. Given the conserved reliance on PRF across the order, we propose that SHFL—which inhibits PRRSV by targeting −1 PRF—may possess broad-spectrum activity against all nidoviruses. This hypothesis is supported by prior studies showing that SHFL significantly restricts the replication of SARS-CoV-2 (21), which belongs to a distinct family within the *Nidovirales*. However, the precise antiviral mechanisms of SHFL appear to involve virus-specific conditions and may extend beyond PRF interference. This is further illustrated by studies in the Flaviviridae family, where SHFL also exhibits broad-spectrum inhibition against various flaviviruses (50), though mechanistic details reveal subtle differences in its mode of action against individual members. Thus, while targeting PRF provides a common and potent basis for suppression across related virus groups, the complete antiviral profile likely combines conserved and virus-specific interactions. In the case of PRRSV, SHFL not only interfered with PRRSV ribosomal frameshifts but also affected RNA translation (Fig. 5I and J). In the dengue virus, SHFL interferes with RNA translation by associating with cellular mRNA-binding proteins and viral RNA (33), suggesting that SHFL may employ a similar mechanism to target PRRSV RNA translation. The detailed mechanism will be further investigated in our future study.

Our data indicate that SHFL employs distinct mechanisms to inhibit the canonical −1 PRF and the novel −2 PRF of PRRSV. Previous studies have identified the amino acids 164–199 region of SHFL as a critical domain for suppressing classical −1 PRF of HIV and JEV (30, 36). In line with those reports, we found that the same region is essential for the inhibitory effect of SHFL on −1 PRF of PRRSV (Fig. 8). In contrast, suppression of −2 PRF requires the full structural integrity of SHFL (Fig. 9F and G), pointing to a different mode of action. The established model for SHFL action against −1 PRF involves its recruitment to stalled ribosomes and binding to −1 PRF RNA, facilitated by a hyper-rotated ribosomal conformation that brings proteins uL5 and eS31 into proximity. Subsequent recruitment of the eRF1/3 complex then leads to premature translation termination (51). However, this model has limitations; for instance, endogenous interactions between SHFL and uL5/eS31 have not been detected (30). SHFL is a strong but relatively nonspecific RNA-binding protein and shows no marked preference for canonical −1 PRF signals or associated downstream structural elements (44). This general RNA-binding activity may also contribute to its inhibition of the novel −2/−1 PRF. Recently, a novel domain within SHFL—designated the HPL domain (amino acids 249–256)—has been identified. Phosphorylation within this domain regulates RNA binding and is important for SHFL-mediated inhibition of −1 PRF (52), suggesting that SHFL phosphorylation may play a key role in regulating both canonical and novel PRF events in PRRSV. We therefore speculate that SHFL may inhibit −2 PRF by directly binding RNA, similar to its action on −1 PRF. Nevertheless, the underlying mechanisms likely differ owing to a key distinction in the downstream regulatory elements: while −1 PRF depends on an RNA pseudoknot, −2/−1 PRF is driven by a protein complex composed of nsp1β and PCBP2. In this context, SHFL could competitively displace the nsp1β/PCBP2 complex from the frameshift RNA sequence, representing a novel, target-specific antiviral mechanism distinct from its action on −1 PRF. We therefore propose a distinct working model for −2 PRF inhibition: SHFL, requiring its full structural integrity, may competitively bind to the −2 PRF RNA sequence, thereby displacing the nsp1β/PCBP2 protein complex. This mechanism, based on “protein complex displacement,” represents a novel, target-specific antiviral action of SHFL that is distinct from its classical mode of inhibiting −1 PRF. However, this working model remains to be further validated experimentally.

Although dual-luciferase assays confirmed that SHFL inhibits both canonical −1 PRF (Fig. 7E through K) and the novel −2 PRF (Fig. 9D through G), this inhibitory effect was not pronounced during actual viral infection. Specifically, the nsp4-to-nsp9 ratio—representing −1 PRF efficiency—remained largely unchanged upon SHFL knockdown (Fig. 2C and 3C). Similarly, −2 PRF efficiency in the context of infection showed only a modest reduction (Fig. 9A and B). Because PRF events generally occur early in PRRSV infection and the infection process itself is dynamic, these observations suggest that the role of SHFL during PRRSV infection is complex and may involve mechanisms beyond direct modulation of PRF. The continuous synthesis and turnover of PRF-derived products throughout the infection course could obscure the inhibitory effect of SHFL at the specific time points examined. Moreover, differences in detection sensitivity—luciferase assays being considerably more sensitive than Western blot—may further limit the ability to capture subtle changes in PRF efficiency during infection. Collectively, these considerations provide a more nuanced interpretation of our data and highlight the inherent challenges in detecting dynamic shifts in PRF efficiency within the context of a live viral infection.

PRRSV also evolves strategies to evade the antiviral effect of SHFL. In PRRSV-infected cells, the inhibitory effect on SHFL transcription was gradually increased. To identify viral proteins that regulate SHFL transcriptionally, we performed a cotransfection assay in which the SHFL promoter was cotransfected with plasmids encoding individual PRRSV proteins (Fig. 12C through E). Nsp12 and N protein significantly inhibited SHFL promoter activity, while nsp1α, nsp1β, nsp2TF, nsp2N, and nsp4 also exerted modest inhibitory effects. A prior report identified a functional nuclear localization signal (NLS) and faster nuclear import relative to export, accounting for its distribution between the cytoplasm and the nucleolus (49). Previously, nsp12 was reported to localize in the cytoplasm and accumulate around the nucleus in punctate and perinuclear spots (53). In this study, we further analyzed the subcellular localization of nsp12 and N in PRRSV-infected cells. In line with previous reports, their presence in both the nucleus and the cytoplasm was determined by IFA and nucleo-cytoplasmic fractionation. In light of these observations, the nuclear localization of nsp12 and N could be critical for their inhibitory effect on SHFL expression at the transcription level. Since the PRRSV life cycle has no nuclear phase, the nuclear import of nsp12 and N should not be required for viral infection. Thus, nsp12 and N could be good targets for the manipulation of the PRRSV genome using a reverse genetics system to inactivate the evasion of SHFL restriction.

In summary, our study demonstrates that SHFL inhibits PRRSV by suppressing viral RNA translation and replication, mechanistically through modulation of −1 PRF and the novel −2 PRF. Notably, we identify the viral proteins nsp12 and N as antagonists of SHFL transcription, counteracting its antiviral effect. Further investigation into SHFL and its viral antagonists will deepen our understanding of host-pathogen interactions during PRRSV infection and contribute to the future development of broad-spectrum antiviral inhibitors.

## Data Availability

All data related to this study are available in the paper.

## SUPPLEMENTAL MATERIAL

Figure S1 jvi.00119-26-s0001.docx Conservation analysis of the PRRSV -1 PRF signal.

ASM does not own the copyrights to Supplemental Material that may be linked to, or accessed through, an article. The authors have granted ASM a non-exclusive, world-wide license to publish the Supplemental Material files. Please contact the corresponding author directly for reuse.
