# Supplementary material for: Porcine reproductive and respiratory syndrome virus antagonizes the host restriction factor SHFL to sustain viral programmed ribosomal frameshifting and replication
Source: J Virol. 2026 Apr 21;100(5):e00119-26. doi: 10.1128/jvi.00119-26 (PMC13185607; doi:10.1128/jvi.00119-26)

**Figure S1. Conservation analysis of the PRRSV –1 PRF signal.** A comparative analysis of the −1 PRF signal sequences within the ORF1a/1b overlap across all sequences derived from 999 PRRSV-2 strains (A) and 530 PRRSV-1 strains (B) available in the NCBI GenBank database (as of June 2024) revealed a remarkably high degree of conservation.


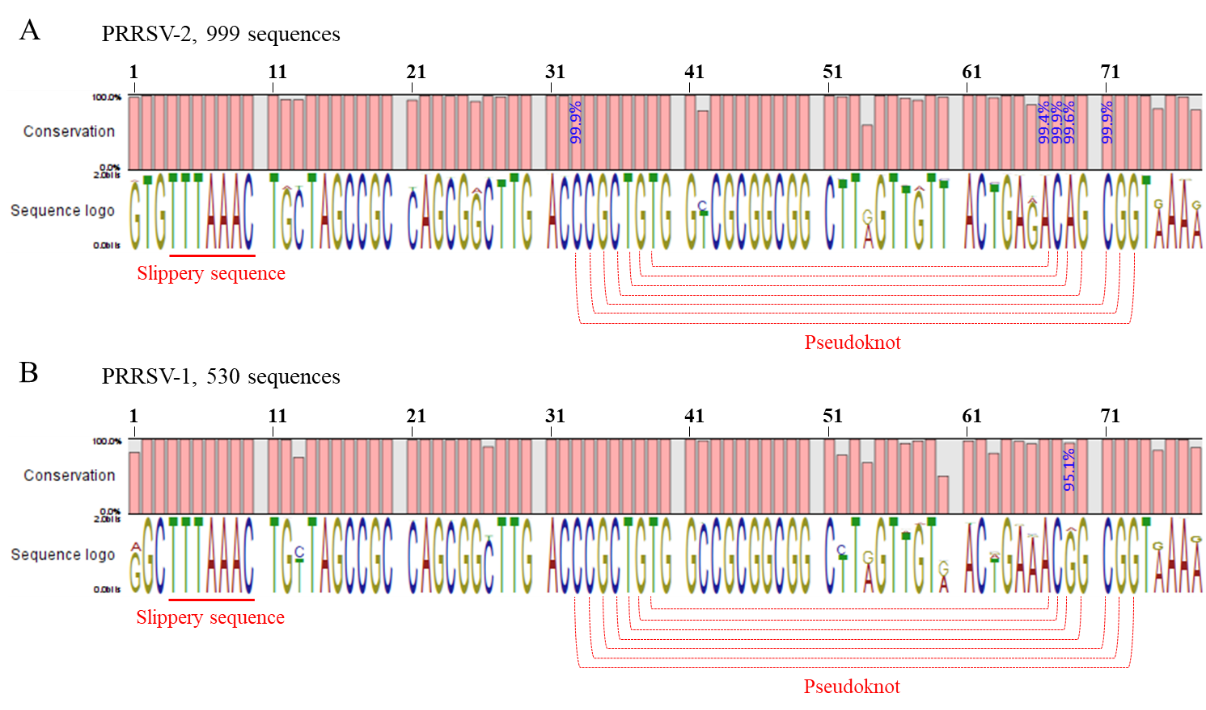

Supplement: Figure S1 — Conservation analysis of the PRRSV -1 PRF signal. [file jvi.00119-26-s0001.docx]
